# Longitudinal Profiles of Dietary and Microbial Metabolites in Formula- and Breastfed Infants

**DOI:** 10.3389/fmolb.2021.660456

**Published:** 2021-05-28

**Authors:** Nina Sillner, Alesia Walker, Marianna Lucio, Tanja V. Maier, Monika Bazanella, Michael Rychlik, Dirk Haller, Philippe Schmitt-Kopplin

**Affiliations:** ^1^ Research Unit Analytical BioGeoChemistry, Helmholtz Zentrum München, Neuherberg, Germany; ^2^ ZIEL Institute for Food and Health, Technical University of Munich, Freising, Germany; ^3^ Chair of Nutrition and Immunology, Technical University of Munich, Freising, Germany; ^4^ Chair of Analytical Food Chemistry, Technical University of Munich, Freising, Germany

**Keywords:** infant, feces, metabolomics, infant formula, bifidobacteria, probiotics, formula-fed, breastfed

## Abstract

The early-life metabolome of the intestinal tract is dynamically influenced by colonization of gut microbiota which in turn is affected by nutrition, i.e. breast milk or formula. A detailed examination of fecal metabolites was performed to investigate the effect of probiotics in formula compared to control formula and breast milk within the first months of life in healthy neonates. A broad metabolomics approach was conceptualized to describe fecal polar and semi-polar metabolites affected by feeding type within the first year of life. Fecal metabolomes were clearly distinct between formula- and breastfed infants, mainly originating from diet and microbial metabolism. Unsaturated fatty acids and human milk oligosaccharides were increased in breastfed, whereas Maillard products were found in feces of formula-fed children. Altered microbial metabolism was represented by bile acids and aromatic amino acid metabolites. Elevated levels of sulfated bile acids were detected in stool samples of breastfed infants, whereas secondary bile acids were increased in formula-fed infants. Microbial co-metabolism was supported by significant correlation between chenodeoxycholic or lithocholic acid and members of Clostridia. Fecal metabolites showed strong inter- and intra-individual behavior with features uniquely present in certain infants and at specific time points. Nevertheless, metabolite profiles converged at the end of the first year, coinciding with solid food introduction.

## Introduction

Nutrition during the early postnatal life is an important factor that might influence health status throughout the whole life (Robinson, 2015). Breast milk is the recommended feeding during the first six months, as pointed out by the world health organization (World Health Organization, 2003). From a nutritional point of view breast milk contains an appropriate composition of macronutrients and micronutrients, important for the child’s development. Macronutrients include carbohydrates (lactose, oligosaccharides), fat (triglycerides with saturated and polyunsaturated fatty acids) and proteins (casein or whey proteins such as α-lactalbumin, lactoferrin, secretory IgA, and serum albumin) (Prentice, 1996). There is a substantial variation of breast milk components between mothers depending on diet and age. Infant formula is an industrially produced alternative to breast milk and is used either as a sole food source or to complement breast milk in early life. Infant formulas are based on cow milk, soy or meet special requirements such as hypoallergenic formulas (Koletzko et al., 2005; Martin et al., 2016a). Feeding of infants with breast milk or formula is discussed to contribute to different outcomes in health and disease (van den Elsen et al., 2019). The impact of feeding type in early-life gained more interest in various omics fields such as metagenomics or metabolomics (Stewart et al., 2018).

A non-targeted metabolomics approach using LC-MS based techniques can be utilized to study diet-related differences between breast- and formula-fed infants. Urine or fecal samples are considered as non-invasive matrices to profile metabolites in health-disease related issues (Wild et al., 2019). Stool metabolites can reflect changes of gut microbial metabolism (Zierer et al., 2018). Additionally, excreted host derived metabolites or digested food ingredients provide insights into non-microbial metabolism.

In previous work, we are able to show that the fecal metabolome and gut microbiome is altered between breast- and formula-fed infants in the first year of life with converging profiles at the age of 12 months (Bazanella et al., 2017). Probiotic supplementation with bifidobacteria showed only minor effects on both, fecal microbiome and metabolome. Despite the identification of short chain fatty acids, only a shallow description of metabolites was given, with few mass signals and the respective annotated classes (Bazanella et al., 2017). Here, we extend our non-targeted metabolomics approach by combining different chromatographic approaches to analyze polar and semi-polar metabolites in infant stool and formula or breast milk, and supporting our metabolite identities with tandem MS based fragmentation experiments and authentic standard matching. The detailed examination between breast- and formula-fed infants allowed us to discriminate between nutrition or microbe derived alterations in the fecal metabolome. Metabolite profiles were monitored at several time points during the first year of life, including a follow-up study one year later, enabling a longitudinal comparison of infant diets.

## Materials and Methods

### Study Design

Stool samples (*n* = 244, Suppementary Table S1) from healthy infants, who received infant formula with (*n* = 11, F+), without (*n* = 11, F−) bifidobacteria (*B. bifidum*, *B. breve*, *B. infantis*, *B. longum*) or exclusively breast milk (*n* = 20, B) were collected over a period of two years in a randomized, double-blinded, placebo-controlled intervention trial as described elsewhere (Bazanella et al., 2017). The samples are a subset of the original study and only infants, who were exclusively fed with breast milk or formula (F− or F+) were taken into the metabolomics study. Fecal samples of month 1, 3, 5, 7, 9, 12 and 24 were selected for non-targeted analysis.

Additionally, breast milk samples (*n* = 42) were collected at different time points and categorized in samples from secretor (*n* = 20) and non-secretor mothers (*n* = 4) (Bazanella et al., 2017). Furthermore, mother-child and time matching infant fecal samples (*n* = 39) were selected for comparison of HMO levels between breast milk and associated infant feces. The trial was registered at the German Clinical Trials Register (DRKS00003660) and the protocol was approved by the ethics committee of the medical faculty of the Technical University of Munich (approval number 5324/12).

### Chemicals

Arachidonic acid, eicosapentaenoic acid, myristic acid, 4-hydroxyphenyllactic acid, indolelactic acid, phenyllactic acid, 6′-sialyllactose, chenodeoxycholic acid (CDCA), cholic acid (CA), ursodeoxycholic acid (UDCA), lithocholic acid (LCA), glychochenodeoxycholic acid (GCDCA) and taurochenodeoxycholic acid (TCDCA) were purchased from Sigma-Aldrich (St. Louis, United States). 7-oxoLCA, 3-dehydroCDCA, 7,12-dioxoLCA, 7-oxodeoxycholic acid (7-oxoDCA), 3-dehydroCA, 7-epiCA, glycocholic acid (GCA) and taurocholic acid (TCA) were purchased from Steraloids (Newport, RI, United States). Cholic acid 7-sulfate (CA-S) and 3′-sialyllactose were purchased from Cayman (Biomol GmbH, Hamburg, Germany). Sulfate (S) conjugates of CDCA, UDCA, and LCA were synthesized according to Donazzolo et al. (Donazzolo et al., 2017) and structures were verified by MS/MS and NMR spectroscopy.

Milli-Q water (18.2 MΩ) was derived from a Milli-Q Integral Water Purification System (Billerica, MA, United States). Acetonitrile (ACN; LiChrosolv®, hypergrade for LC-MS), methanol (LiChrosolv®, hypergrade for LC-MS) and ammonium acetate (NH_4_Ac) were obtained from Merck (Darmstadt, Germany). Glacial acetic acid was purchased from Biosolve (Valkenswaard, Netherlands) and formic acid from Honeywell Fluka™ (Morristown, NJ, United States).

### Fecal Sample Preparation

Metabolite extraction from infant stool samples was prepared with methanol as described previously (Bazanella et al., 2017). For hydrophilic interaction liquid chromatography (HILIC) analysis the methanol extracts were evaporated under vacuum at 40°C (SpeedVac Concentrator, Savant SPD121P, ThermoFisher Scientific, Waltham, MA, United States) and reconstituted with ACN/H_2_O 75:25 (v/v). A pooled sample was generated from all fecal extracts for quality control purposes. All samples were stored at −80°C in tightly closed tubes.

### Breast Milk Sample Preparation

For HILIC measurements, 125 µL breast milk was extracted with 375 µL acetonitrile. The mixture was vortexed, centrifuged with 14,000 rpm at 4°C for 10 min and the supernatant was collected. A pooled sample was generated from all breast milk extracts for quality control purpose. All samples were stored at −80°C in tightly closed tubes.

### Quantification of Fatty Acids in Formula and Breast Milk

Breast milk samples from lactation month 1 (*n* = 26), month 3 (*n* = 28) and month 4 (*n* = 9) were pooled, respectively. Additionally, three types of infant formula (pre, 1 and 2), consumed by infants of this study, were mixed with hot tap water (∼50°C) according to manufacturer instructions. The method for quantification of the fatty acids in the different milk samples was described by Firl et al. (Firl et al., 2014).

### HILIC UHPLC-MS/MS Screening

The fecal and breast milk extracts as well as standard substances (in ACN/H_2_O 75:25, v/v) were analyzed by UHPLC (Acquity, Waters, Milford, MA, United States) coupled to a time of flight (TOF) mass spectrometer (MS) (maXis, Bruker Daltonics, Bremen, Germany). HILIC was performed using an iHILIC®-Fusion UHPLC column SS (100 × 2.1 mm, 1.8 µm, 100 Å, HILICON AB, Umea, Sweden). Chromatographic settings were the same as previously described (Sillner et al., 2019a) with the following modifications: injection volume was 5 µL, eluent A consisted of 5 mmol/L NH_4_Ac (pH 4.6) in 95% ACN (pH 4.6) and eluent B of 25 mmol/L NH_4_Ac (pH 4.6) in 30% ACN with a runtime of 12.1 min, followed by reconditioning for 5 min after each sample, respectively. Every tenth injection a pooled fecal sample was used as quality control for subsequent batch normalization.

Calibration of the MS was done by injecting ESI-L Low Concentration Tuning Mix (Agilent, Santa Clara, CA, United States) prior to the measurements. Additionally, ESI-L Low Concentration Tuning Mix (diluted 1:4 (v/v) with 75% ACN) was injected in the first 0.3 min of each UHPLC-MS/MS run by a switching valve for internal recalibration. Mass spectra were acquired in negative electrospray ionization mode (-ESI), respectively. Parameters of the ESI source were: nitrogen flow rate: 10 L/min, dry heater: 200°C, nebulizer pressure: 2 bar and capillary voltage: 4,000 V. Data were acquired in line and profile mode with an acquisition rate of 5 Hz within a mass range of 50–1,500 Da. Data-dependent MS/MS experiments were performed in automated MS/MS mode. After each precursor scan, the five most abundant ions (absolute intensity threshold ≥ 2,000 arbitrary units) were subjected to MS/MS. Each fecal sample was measured in duplicates with a collision energy of 10 and 35 eV, respectively.

The RP UHPLC-MS measurements for non-targeted metabolomics and short chain fatty acid analysis are described elsewhere (Bazanella et al., 2017). Originally, no data dependent MSMS experiments were performed for the RP UHPLC-MS analysis of 244 fecal samples. Data dependent MSMS experiments were done on selected (pooled) samples or targeted MSMS or authentic standard matching (bile acids and fatty acids) for verifying significant RP UHPLC-MS features.

### Data Processing

Raw UHPLC-MS and UHPLC-MS/MS data were processed with Genedata Expressionist Refiner MS 11.0 (Genedata GmbH, Munich, Germany), including chemical noise subtraction, intensity cut-off filter, calibration, chromatographic peak picking and deisotoping. Metabolite library search and classification was done with the Human Metabolome Database (HMDB) (Wishart et al., 2018) for MS1 (±0.005 Da). Spectral library matching was done with MS PepSearch (0.01 Da for Precursor and Fragment Match) and matches above dot product of 500 were kept. Additionally, matches with ion charge discrepancies (+1) of library hits were removed. Theoretical mass signal values were calculated for all found metabolites. These theoretical mass signals were compared to library precursor mass signals and only metabolite library matches with error <0.01 Da were kept. Metabolite matches with higher dot product were kept if clusters had multiple matches in spectral libraries. All spectral libraries were downloaded from MassBank of North America (https://mona.fiehnlab.ucdavis.edu/) to perform MS/MS matching. Human milk oligosaccharides were identified by matching against two mass spectral reference libraries (Human Milk SRM1953 and Milk_OIigosaccharide_MS_library_2019), reported here (Remoroza et al., 2018; Remoroza et al., 2020). Manual classification and identification were done for Maillard reaction products. We reported metabolites as following: Level 3 are metabolites that are identified based on MS1 annotation and Level 2 are metabolites that are identified with the help of spectral library matching, authentic standard matching or manual inspection and evaluation (for example applicable for Maillard reaction products) (Sumner et al., 2007). Peak areas of duplicates were averaged and normalized to fecal weight (wet weight). Batch normalization based on consecutive quality control measurement samples (pooled sample) was performed after missing value imputation (randomized number between 1.0 and 1.2, based on lowest value of the data matrix). Targeted MS/MS experiments of Amadori products were performed in multiple reaction monitoring mode (MRM) at 20 eV in positive ionization mode.

### Statistical Analysis

Multilevel partial least squares discriminant analysis (PLS-DA) was applied to consider the paired and multi-factorial (feeding type and time) design of the study. The validity of the multilevel PLS-DA model was confirmed by 7-fold cross validation and receiver operating characteristic (ROC) curves, by evaluating the first and second component. Univariate statistical analysis was done by calculating *p*-values with Kruskal-Wallis rank sum test (for multi-group comparison) or Wilcoxon rank sum test (two group comparison) using the ggpbur package of the R environment. Regularized canonical correlation analysis (rCCA) was applied for correlation between OTU (operational taxonomic unit) and bile acid data (Meng et al., 2014). The vertical integration method combined the two datasets (Sperisen et al., 2015). For the hierarchical clustering of bile acids and OTUs, we used Euclidian distance and the average agglomeration method and relevance networks were applied as described here (González et al., 2012). The significant interaction between bile acids and OTUs was determined by calculating pairwise Spearman rank correlation values and their respective *p*-values. *p*-values were corrected for multiple comparisons according to the Benjamini-Hochberg method. Inter- and intra-individual metabolites were selected by multiple co-inertia analysis, available at the omicade4 package of R environment (Meng et al., 2014). Prior to multivariate analyses, the data was log-transformed, and unit-variance scaled.

### High-Throughput 16S rRNA Gene Sequencing

Samples were prepared and analyzed as described previously (Bazanella et al., 2017). In brief, 100 mg was used to extract DNA for metagenomics analysis. DNA stabilization buffer (600 μL) and 400 μL phenol/chloroform/isoamyl alcohol (25:24:1, v:v; Sigma-Aldrich) was added to the samples. Disruption of cells was performed with a bead beater and heat treatment (95°C, 8 min), followed by centrifugation and incubated with ribonuclease. DNA concentration was determined with NanoDrop. The V3-V4 region was sequenced by a MiSeq system (Illumina Inc.). Raw data was processed as following. The reads were processed in an in-house developed pipeline Integrated Microbial Next Generation Sequencing (Lagkouvardos et al., 2016). Sequences were demultiplexed, trimmed and paired. Operational taxonomic units (OTUs) were clustered at 97% sequence similarity, and OTUs with a relative abundance >0.5% in ≥1 sample were kept. Taxonomies were assigned at 80% confidence level with the RDP classifier (Wang et al., 2007), as described in previous publication. OTU table was normalized to adjust different sequence depths by division to their sample size and then multiplication by the size of the smaller sample. OTU data was used for multivariate and univariate integration and correlation analysis. Raw sequence data are available at the European Nucleotide Archive under study accession number ERP023432.

## Results and Discussion

### Time Independent Feeding Effects on the Fecal Metabolome

**FIGURE 1:**
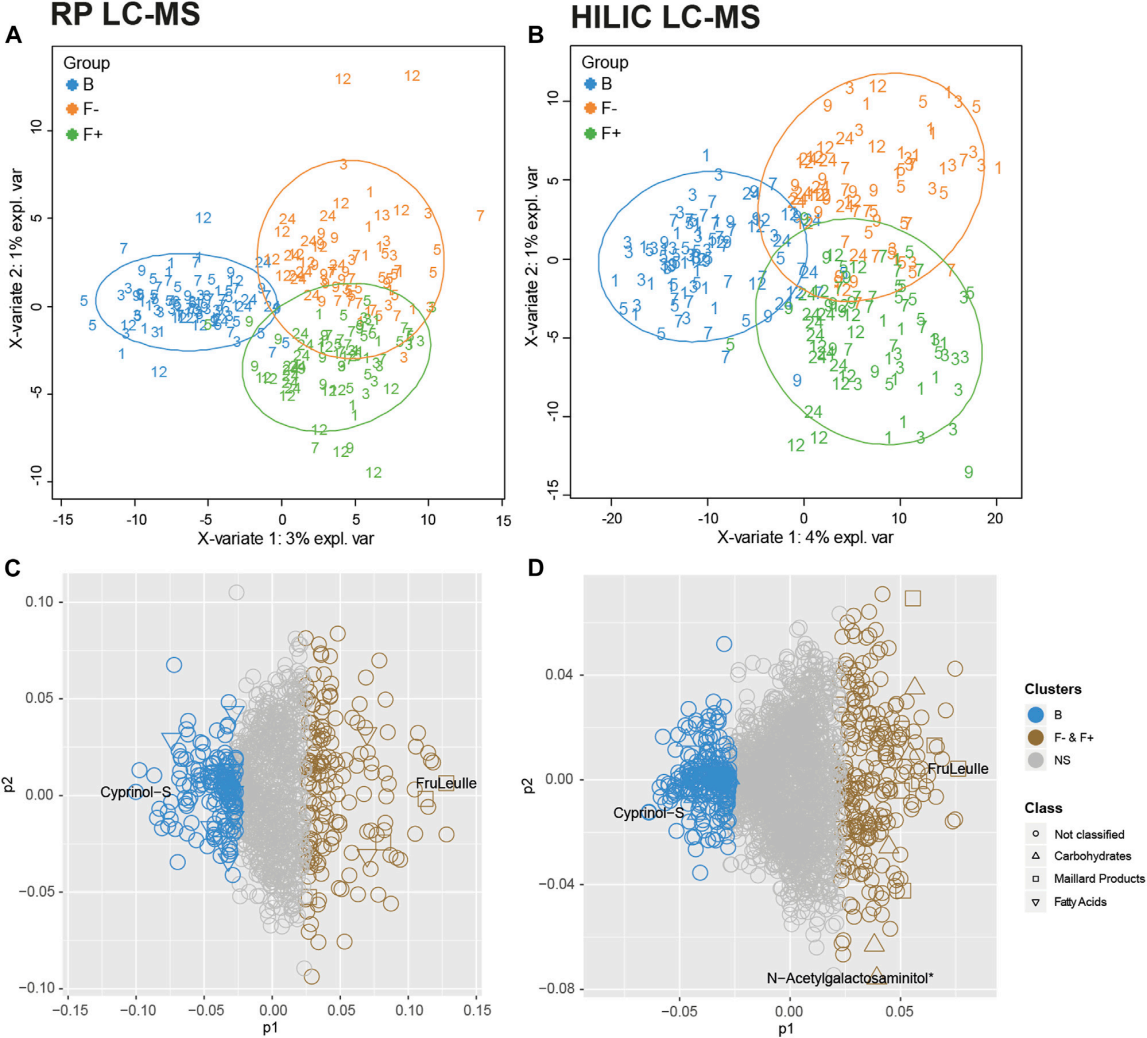
Multilevel PLS-DA. Scatter plots of **(A)** RP—and **(B)** HILIC-UHPLC-MS measurements (negative electrospray ionization mode). Groups were separated according to feeding and corrected for time. Numbers represent age of infants at the time of feces sampling (month 1–12, 24). Multilevel PLS-DA loading plots for **(C)** RP and **(D)** HILIC UHPLC-MS. Top 15% discriminant features were highlighted for B (breastfed, blue) or both F groups (formula-fed, brown). The remaining features are presented in grey (NS = not significant). Putative carbohydrates (open triangle), fatty acids (open inverted triangle) and Maillard products (open box) are representing significant clusters between B and F groups. Cyprinol-S (-sulfate) was increased in breastfed infants, the Amadori product FruLeuIle (*N*-deoxyfructosylleucylisoleucine) was specifically representing both F groups. N-Acetylgalactosaminitol was increased in the F + group. *detected as [M + Cl]^−^ adduct.

The particular design of the infant study with longitudinal sampling requires appropriate statistical methods due to high dimensional and co-linear metabolite data. We apply a multilevel PLS-DA model to analyze the feeding effect on paired biological samples at different time points. The first component of the multilevel PLS-DA explained 4% for RP dataset (Figure 1A) and 3% for HILIC dataset (Figure 1B) of the total variance between B and F groups. Despite the clear visual separation of feeding groups, the relatively low percentage of the feeding variable on the total variance can be explained by the fact that infant fecal metabolomes are influenced by many different variables such as the amount of food, maternal diet of breastfed children, gastrointestinal passage or digestion status and sampling time, all contributing to the variability. Probiotic treatment (F^+^, green) could explain only 1% of the total variance in the metabolite data. Only few infants of the F^+^ group in month 1 and 3 responded to probiotics, as seen in the right lower corner of Figures 1A,B. Receiver Operator Characteristic (ROC) curves of the multilevel PLS-DA component 1 confirmed with around 0.99 classification accuracy that B are highly distinct from F groups (Supplementary Figures S1A,C). Probiotic supplemented vs. non-supplemented formula feeding, represented in component 2 (Figure 1), resulted in slightly lower classification accuracy of around 0.98 (Supplementary Figures S1B,D). For further investigations, the most contributing features (top 15%) were selected from the loadings plot, starting from lowest (B) or highest (F− and F+) principal component 1 (p1) coordinates and the same for principal component 2 (p2) coordinates (F− vs. F^+^) values (Figures 1C,D).

Putative annotation of features indicated that group B is described by “*fatty acids and conjugates*” for RP and “*carbohydrates and carbohydrate conjugates*” for HILIC analysis, whereas many characteristic metabolites of both F groups turned out to be Maillard reaction compounds, especially Amadori products (Sillner et al., 2019b). During the identification process, features were grouped in either nutrition or microbe derived metabolites, based on existing literature (Bode, 2012; Pischetsrieder and Henle, 2012; Dodd et al., 2017; Sillner et al., 2018; Pranger et al., 2019). Metabolites were annotated (level 3) or identified (level 2). The list of all features of the RP and HILIC analysis including the loadings information is available in Supplementary Table S1. Several studies consistently showed that children fed with breast milk or formula have a clear and distinct fecal metabolome, as recently summarized by Laurens et al. (Laurens et al., 2020). Targeted or non-targeted profiling of infant stool resulted in the detection of various metabolite classes including short chain fatty acids (SCFAs), amino acids, amino acid catabolites, fatty acids, human milk oligosaccharides, monosaccharides, purine degradation products or even plant-based metabolites from soy-based formula (Chow et al., 2014; Martin et al., 2014; Dotz et al., 2015; Dotz et al., 2016; Martin et al., 2016b; Bridgman et al., 2017; He et al., 2019; Brink et al., 2020; Kok et al., 2020; Li et al., 2020).

### Feeding Type-Derived Metabolites Over Time

#### Increased Fatty Acids in Breastfed Children Reflected Fatty Acid Composition of Breast Milk

**FIGURE 2:**
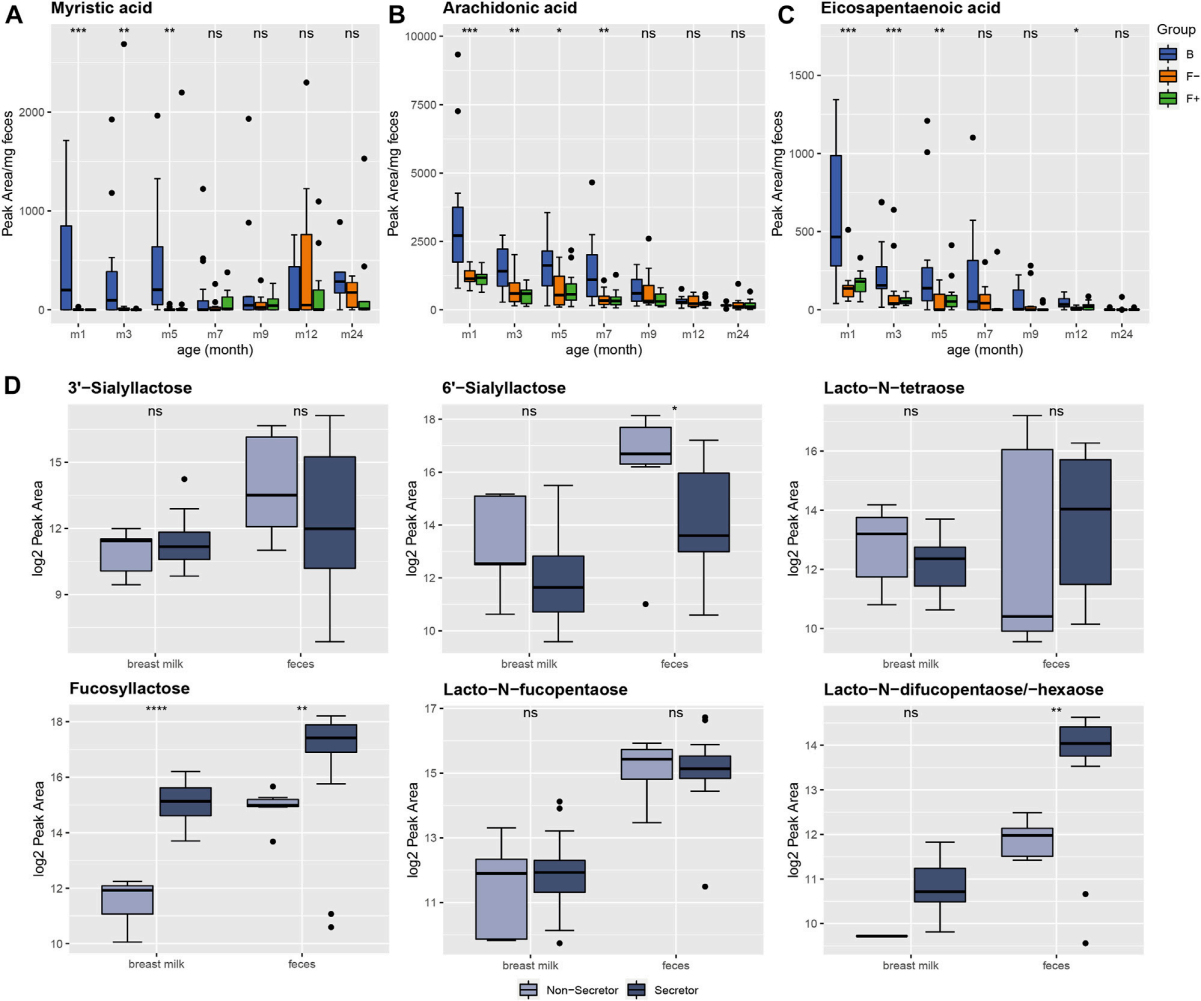
Diet-related alteration of fatty acid profiles in feces of breastfed (B, blue) and formula-fed infants without (F-, orange) or with probiotics (F^+^, green) over time. **(A)** Myristic, **(B)** arachidonic and **(C)** eicosapentaenoic acid were increased in group B up to month 7. Peak areas exceeding 10*mean for individual fatty acid were excluded from plotting procedure for a better visualization. **(D)** Detection of six different human milk oligosaccharides (HMOs) in breast milk and infant feces. Breast milk samples were categorized in secretor (*n* = 35, dark-blue) and non-secretor (*n* = 7, light-blue). Breast milk (*n* = 42) and feces samples (*n* = 39) were mother-child and time (m1, 3) matched for comparison. The secretor status determined the relative amount of different HMOs in breast milk as well as in the corresponding feces samples. Significance was calculated in each month between B, F− and F+ group using the Kruskal-Wallis rank sum test or Wilcoxon rank sum test for comparing secretor and non-secretor status. ns: *p* > 0.05, *p ≤ 0.05, **p ≤ 0.01, ***p ≤ 0.001, ****p ≤ 0.0001.

The semi-polar fecal metabolome of group B was discriminated by the subclass of fatty acids and conjugates, as described above. In feces of breastfed infants, the saturated fatty acid myristic acid (C14:0) was elevated from month (m) 1–5. In formula-fed infants myristic acid was very low abundant during the first 3 months but showed very similar levels to breastfed infants at month 12 and 24 (Figure 2A), probably due to the predominant solid food nutrition in all of the groups at this age. Myristic acid was also reported to be higher in cecal contents of breastfed piglets (Poroyko et al., 2011). Several free fatty acids including myristic acid, palmitic acid, linoleic acid, linolenic acid, oleic acid, and palmitoleic acid were increased in large intestine contents of piglets fed with human milk, which is in line with our results (Rosa et al., 2020). On the contrary, a different study reported myristic acid to be significantly higher in formula-fed infants (Chow et al., 2014). Li et al. observed a series of different fatty acids, significantly altered in fecal samples of breastfed, mixed-fed, exclusively formula-fed or complementary food-fed infants (Li et al., 2020). In the mixed-fed group they detected increased levels of cis-11,14-eicosadienoic acid, stearidonic acid, capric acid, myristic acid, docosahexaenoic acid, cis-8,11,14-eicosatrienoic acid, and 15-oxoete. Interestingly, only eicosapentaenoic acid was elevated in stool samples of the exclusive formula-fed group (Li et al., 2020). Varying results of fecal fatty acid profiles between different studies could be due to the variable fatty acid composition of different infant formulas. To verify our findings, we quantified the fatty acids in breast milk and infant formula relevant for our study (Supplementary Table S2). Indeed, the average concentration of myristic acid in breast milk was found to be much higher than in the infant formulas (pre, 1, 2) consumed by our cohort. Furthermore, also the long chain poly-unsaturated fatty acids arachidonic (Figure 2B) and eicosapentaenoic acid (Figure 2C) were increased in breastfed infants compared to the two formula-fed groups up to month 7. Again, this relation was confirmed in the fatty acid analysis of breast milk and formula (Supplementary Table S2), showing higher concentrations of arachidonic and eicosapentaenoic acid in breast milk. Although, the total amount of poly-unsaturated fatty acids was higher in formula. The fatty acid profile in breast milk can be influenced by the mother’s diet (Xiang et al., 2005; Kim et al., 2017). However, correlation analysis between mother’s diet and fatty acid profiles in breast milk was not applicable, since no information about mother’s diet was collected during this study.

#### Infant Fecal Human Milk Oligosaccharides Reflected the Secretor Status of the Mothers

In the HILIC analysis, human milk oligosaccharides (HMOs) were very characteristic for the fecal metabolome of breastfed infants. HMOs are only present in human breast milk, therefore these mass signals were absent in the formula-fed group. The HMO profile in breast milk is determined by the secretor status. Non-secretor mothers have an inactive allele of the maternal fucosyltransferase 2 (FUT2) gene and therefore can’t produce α1-2 fucosylated HMOs, e.g., 2′-fucosyllactose. According to this, breast milk samples were classified in secretor (*n* = 35) and non-secretor (*n* = 7) samples by Bazanella et al. (Bazanella et al., 2017). This classification was also done for mother-child matched fecal samples to examine the influence of the mother’s secretor status on the HMO profiles in feces of their infants by applying a non-targeted profiling HILIC-MS approach on both matrices. Therefore, we analyzed fecal (*n* = 39) and breast milk samples (*n* = 42) collected in different months (m1 and m3). In total, we detected six different HMOs (Figure 2D; Supplementary Table S3). Unfortunately, no differentiation between 2′-fucosyllactose (2-FL) and 3′-fucosyllactose (3-FL) was possible with the non-targeted screening method, therefore both were summarized as fucosyllactose (Figure 2D). However, the proportion of 2-FL is higher than 3-FL in secretors (Plows et al., 2021). Moreover, 3-FL is not influenced by secretor status (De Leoz et al., 2012), thus, the summed intensities of 2-FL and 3-FL were still representing the secretor status. The relation of the HMOs in breast milk from secretor vs. non-secretors were very precisely reflected in the fecal samples of infants from the corresponding secretor vs. non-secretor mothers (Figure 2D). Interestingly, this was not only the case for fucosylated but also for sialylated HMOs, like 3′- and 6′-sialyllactose (Figure 2D). Identification of HMOs in infant feces was already reported in different studies (Dotz et al., 2015; Dotz et al., 2016) and therefore our results underline that the mothers secretor status can be determined by analyzing HMOs in infant stool, especially by profiling fucosyllactose (Figure 2D). Chow et al. also observed higher levels of 2-FL and lacto-N-fucopentaose in breastfed children. The secretor status influences the infant gut microbiota composition and therefore can impact the fecal metabolome. Feeding with breast milk from secretor mothers enhances the colonization with specific bifidobacteria, which are common infant gut commensals (Lewis et al., 2015; Stewart et al., 2018).

#### Maillard Reaction Products Dominated the Stool Metabolome of Formula-Fed Infants

**FIGURE 3:**
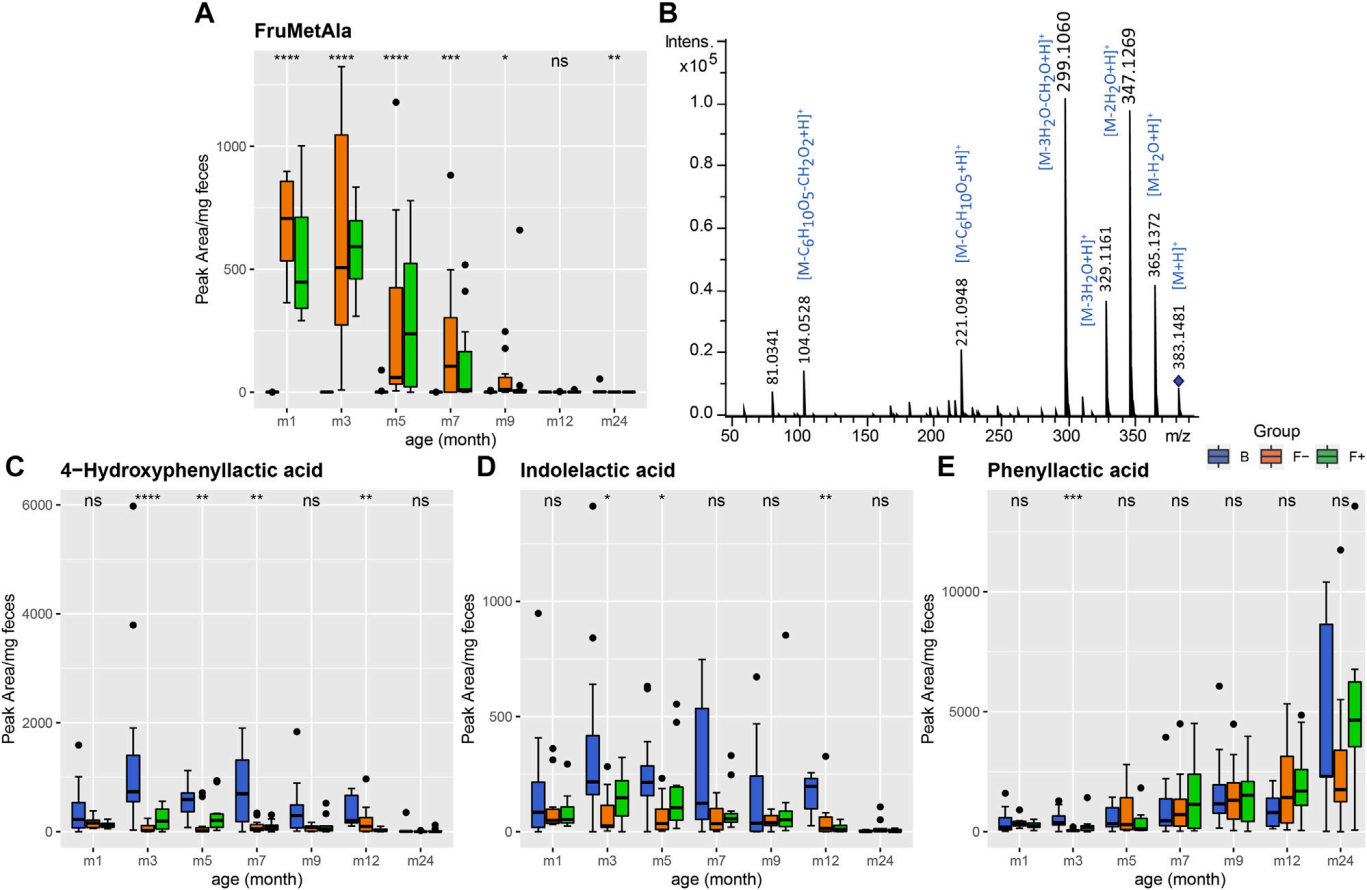
**(A)** Fecal excretion profile of the putative Amadori product FruMetAla during the first 2 years of life. In formula-fed infants (F−, without probiotics, orange and F+, with probiotics, green) the amount of excreted FruMetAla decreased over time. In breastfed infants (B, blue) FruMetAla was not detectable. **(B)** Collision induced dissociation MS/MS experiment (20 eV, positive ionization mode) of FruMetAla in a pooled fecal sample. **(C–D)** Profiles of bacterial aromatic amino acid degradation metabolites in feces of breastfed (group B, blue) and formula-fed infants without (group F−, orange) or with probiotics (group F+, green) over time. **(A)** 4-Hydroxyphenyllactic and **(B)** indolelactic acid were increased in group B up to month 7. **(C)** Fecal phenyllactic acid increased over time in all three groups. Significance was calculated in each month between B, F−, and F+ group using the Kruskal-Wallis rank sum test. ns: *p* > 0.05, *p ≤ 0.05, **p ≤ 0.01, ***p ≤ 0.001, ****p ≤ 0.0001.

Recently, we identified the milk-derived Amadori products *N*-deoxylactulosyl- and *N*-deoxyfructosyllysine and *N*-deoxylactulosyl- and *N*-deoxyfructosylleucylisoleucine in stool from the same cohort, which were only present in formula-fed infants (Sillner et al., 2019b). N-deoxyfructosylleucylisoleucine (FruLeuIle) is also the most discriminating known feature of F group in both datasets (Figure 1A,B). Amadori products are early Maillard reaction products and are formed during infant formula production, mainly between lactose and protein bound amino acids (N-terminal or lysine side chains). We were able to annotate further Amadori products as significant features of metabolome the formula-fed children, due to their characteristic MS/MS patterns (Figure 3B). The *m/z* signal 381.1338 was only present in formula-fed infants and decreased slowly with time, most likely due to solid food introduction (Figure 3A; Supplementary Table S1). Structural identification was done in positive ionization mode, because Amadori products are denser on MS2 fragments. MRM fragmentation in positive mode of the corresponding *m/z* signal 383.1481 is shown in Figure 3B. Dominant fragment ions resulted from neutral losses of water, loss of 84 Da (-3H_2_O-CH_2_O) and loss of the glucose moiety (−162 Da), which are characteristic for the Amadori compound class (Hegele et al., 2008; Wang et al., 2008; Ruan et al., 2018). Accordingly, this metabolite was annotated as *N*-deoxyfructosylmethionylalanine (FruMetAla) due to its exact mass (±0.005 Da mass tolerance), MS/MS fragmentation pattern and the matching N-terminal amino acid sequence MetAla of the formula ingredient glycomacropeptide (Neelima et al., 2013). Glycomacropeptide is released from κ-casein during whey powder production and remains in the sweet whey fraction, which is among others used for infant formula (Rigo et al., 2001).

Furthermore, two features were annotated as *N*-deoxyfructosylacetyllysine (FruAcLys) (Supplementary Figure S2). Their fragmentation patterns are similar, however the earlier eluting peak (6.2 min) exhibits the typical loss for Amadori products of 84 Da (Supplementary Figure S2A), whereas the peak at 6.6 min shares also fragments with *N*-deoxyfructosyllysine (FruLys) (Supplementary Figure S2B) (Sillner et al., 2019b). During the Maillard reaction 1-deoxy-2,3-hexodiulose reacts with lysine and degrades to acetyllysine via β-dicarbonyl cleavage (Smuda et al., 2010; Henning et al., 2011). Since lysine has two amine groups, formation of an acetyllysine Amadori product could be possible during infant formula production. Indeed, we were able to detect peaks with the same mass and retention time in a model reaction mixture of L-lysine with glucose heated for 1 h at 100°C in water, maybe deriving from glycated α- and ε-acetyllysine (Supplementary Figure S2C). FruAcLys and especially FruMetAla could serve as specific nutrition markers for formula-fed infants, similar to the previously proposed compounds called *N*-deoxyfructosyl-/*N*-deoxylactulosylleucylisoleucine (Sillner et al., 2019b). Recently, it has been shown that processed whey protein or FruLys affect gut microbiota with increasing specific species such *Collinsella intestinalis* (Wolf et al., 2019).

### Microbial Derived Metabolites Over Time

#### Feeding Type and Time Dependent Alteration of Microbial Aromatic Amino Acid Metabolites

During the first year, 4-hydroxyphenyllactic and indolelactic acid (Figures 3C,D) were increased in fecal samples of breastfed infants. Although 4-hydroxyphenyllactic and indolelactic acid were not significantly changed between F− and F+, higher intensity levels were observed for the bifidobacteria-supplemented F+ group at month 3 and 5. It was reported that bifidobacteria, which are predominant gut microbes in breastfed children, produce phenyllactic, 4-hydroxyphenyllactic (Beloborodova et al., 2009; Beloborodova et al., 2012) and indolelactic acid (Aragozzini et al., 1979) from the aromatic amino acids phenylalanine, tyrosine and tryptophan, respectively. However, the profiles of the corresponding amino acids did not show any coherent behavior in feces (Supplementary Figure S3), probably due to fluxes into multiple metabolite pathways. Interestingly, phenyllactic acid showed a quite different profile compared to the others with no significant differences between the feeding groups but increasing levels over time (Figure 3E). This may indicate that other bacterial species are settling the children’s gut in the course of time, which are able to produce higher amounts of phenyllactic acid. Whereas 4-hydroxyphenyllactic and indolelactic acid almost disappeared completely after introduction of solid food (24 m). Brink et al. reported three different metabolites of the tryptophan metabolism such as kyneuric acid, indole-3-lactic acid and indole-3-acetic acid to be increased in feces of breastfed infants compared to dairy- and soy-based formula-fed children (Brink et al., 2020).

#### Formula Feeding Increased the Variety of Microbial Secondary Bile Acids

**FIGURE 4:**
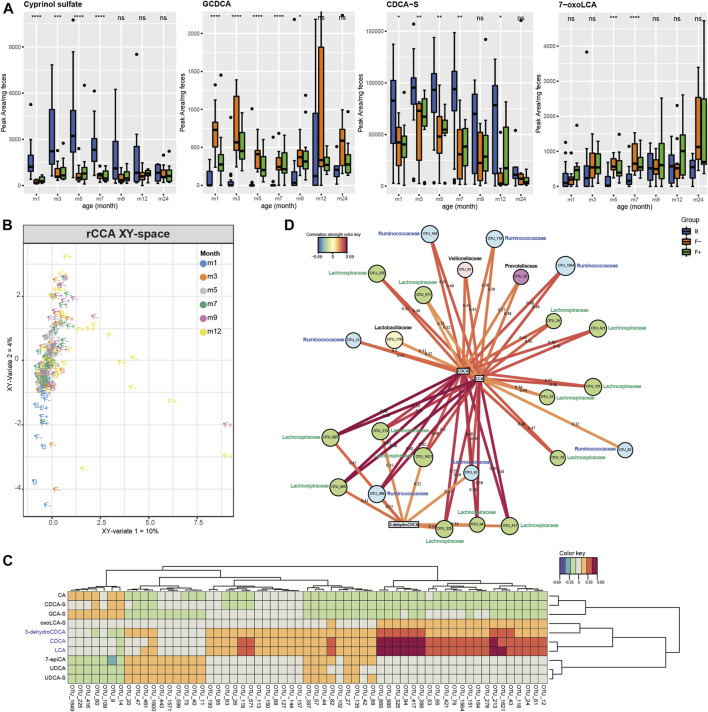
**(A)** Individual boxplots of four bile acids, altered between B, F−, and F+ group in each month. Peak areas exceeding 5*mean for individual bile acids were excluded from plotting procedure for a better visualization. Significance was calculated in each month between B, F−, and F+ group using the Kruskal-Wallis rank sum test. ns: *p* > 0.05, *p ≤ 0.05, **p ≤ 0.01, ***p ≤ 0.001, ****p ≤ 0.0001. Regularized canonical correlation analysis (rCCA) was used to investigate the correlation between two datasets including LC-MS peak area-based bile acid data (X) and OTU data from 16S rRNA sequencing (Y). **(B)** XY-sample plot is colored according the months and labeled according to the feeding type. **(C)** Clustered image map of the cross-correlation matrix revealed a distinct cluster of positively correlating OTUs with LCA, CDCA, and 3-dehydroCDCA. **(D)** Corresponding relevance networks for correlations between the two datasets (XY). OTUs are represented by round and metabolites by rectangular nodes. Edges are correlation values, derived from rCCA. Correlation strength > ± 0.3 represented by edges was found for LCA, CDCA, and 3-dehydroCDCA. Solely positive correlations were found within these limits. Most of the correlating OTUs belong to the families *Lachnospiraceae* (green nodes) and *Ruminococcaceae* (blue nodes), both of them belong to the class of Clostridia.

Furthermore, several fecal bile acids were significantly affected by the different feeding types (Figure 1). We could identify twenty bile acids and annotate the bile acid precursor cyprinol sulfate. We found primary, secondary, glycine-, taurine and sulfate-conjugated bile acids (Figure 4A; Supplementary Figure S4). Moreover, the putative identified cyprinol sulfate was the most important feature in discriminating breastfed group (Figures 1A,B) and it was increased until month seven in B group (Figure 4A). All bile acid profiles are illustrated in Supplementary Figure S4A in a time - and feeding type-dependent heatmap. Feeding type specific patterns were observed for example for, GCDCA, and several sulfated species e. g. CDCA-S (Figure 4A). Interestingly, the common secondary bile acid LCA was almost absent until month 12 (Supplementary Figure S4B). It was already described that the concentration of secondary bile acids are much lower in children compared to adults (Huang et al., 1976) and that LCA producing bacteria (7α-dehydroxylation) are at first established in the gut at the age of 12–18 months (Eyssen, 1973). Hammons et al. were able to detect LCA in some infants already at the age of approximately 3 months but with a high inter-individual variation (Hammons et al., 1988). Instead, other secondary bile acids were detected in the earlier months, namely 7-oxoLCA, 3-dehydroCDCA, UDCA, 7-12-dioxoLCA, 7-oxoDCA, 3-dehydroCA and 7-epiCA (Figure 4A; Supplementary Figures S4A,B). Lester et al. already reported in the past about the presence of “unconventional” bile acids in infant feces, like UDCA (Lester et al., 1983). However, 7-oxoLCA, 3-dehydroCDCA, UDCA and 7-epiCA were higher in the formula-fed groups (F+ and F−) (Figure 4A; Supplementary Figures S4A,B). This could be due to the fact that formula-fed children showed a higher bacterial richness and diversity compared to breastfed (B) (Bazanella et al., 2017). A higher excretion of secondary bile acids in formula-compared to breastfed infants was also reported by Hammons et al. (Hammons et al., 1988). Moreover, in a piglet model study higher fecal concentrations of the secondary bile acids DCA, LCA and HDCA were detected in neonatal dairy- and soy-based formula-fed piglets compared to the sow-fed group (Mercer et al., 2018). In our study, the breastfed group was strongly characterized by sulfated bile acids during the first year. Sulfation of bile acids is a major detoxification pathway in humans, which increases their solubility, decreases intestinal absorption, and enhances fecal and urinary excretion. In human adults, it was reported that more than 70% of bile acids in urine are sulfated, whereas the amount in feces is much lower (Alnouti, 2009). The high amount of sulfated bile acids in feces of breastfed infants could be due to a defense mechanism against bile acid accumulation, which is suggested in cholestatic diseases (Alnouti, 2009), and a decreased absorption rate in the large intestine compared to adults. Heubi et al. reported normal enterohepatic circulation of bile acids to begin at first at the age of 3–7 months (Heubi et al., 1982). The conjugated primary bile acids GCDCA and GCA (Figure 4A; Supplementary Figure S4B) were increased in F- during month 1 and 3. Bifidobacteria show bile salt hydrolase (BSH) activity and deconjugate bile acids (Grill et al., 1995; Tanaka et al., 2000; Kim et al., 2004; Begley et al., 2006; Degirolamo et al., 2014). The intensities of GCDCA and GCA in the probiotic F+ group during month 1 and 3 were lower and more similar to group B, which could be a sign of probiotic activity. In the fecal content of 21 days old piglets only unconjugated bile acids were detected by Mercer et al. (Mercer et al., 2018). However, the total excretion was 14- and 47-fold higher in dairy- and soy-based formula-fed piglets compared to sow-fed, whereas fecal cholesterol concentrations were much lower, suggesting that formula feeding leads to increased CYP7A1 expression and fecal bile acid loss in neonatal piglets. Yet, in our infant study we detected no major differences in unconjugated primary bile acids such as CDCA and CA, but higher levels of the glycine conjugates GCDCA and GCA in formula-fed children (Figure 4A; Supplementary Figure S4B). Additionally, a regularized canonical correlation analysis (rCCA) (González et al., 2008; González et al., 2012) was performed to explore relationships between bile acids and OTU data (Figure 4B) and supported by pairwise Spearman correlations (Supplementary Table S4, Benjamini-Hochberg corrected *p* ≤ 0.05). A representation of variables defined by the first two canonical variates is displayed in Supplementary Figure S5. Based on the clustered image map of the cross-correlation matrix (Figure 4C) relevance networks were generated (Figure 4D). Correlations between OTUs and bile acids with a strength > ± 0.3 were found for CDCA, LCA and 3-dehydroCDCA. Correlation strengths > ± 0.5 were only calculated for CDCA and LCA (Figure 4D). Solely positive correlations were found within these limits. Most of the correlating OTUs belong to the families *Lachnospiraceae* and *Ruminococcaceae*, both belong to the class of Clostridia. It is known that many Clostridia species can produce the secondary bile acid LCA from the primary bile acid CDCA via 7α-dehydroxylation. The secondary bile acid 3-dehydroCDCA is also produced from CDCA but via 3α-hydroxysteroid dehydrogenases, which were detected in Firmicutes (Fiorucci and Distrutti, 2015). Others observed strong positive correlations between duodenal bacteria and taurine- and glycine conjugated bile acids, detected in serum of piglets. In total, 15 different genera correlated significantly with glycohyocholic, taurohyocholic and taurocholic acid, and all genera were increased in sow milk fed piglets (Mercer et al., 2018).

### Distinct Time and Diet Inter-and Intra-individual Metabolite Profiles

**FIGURE 5:**
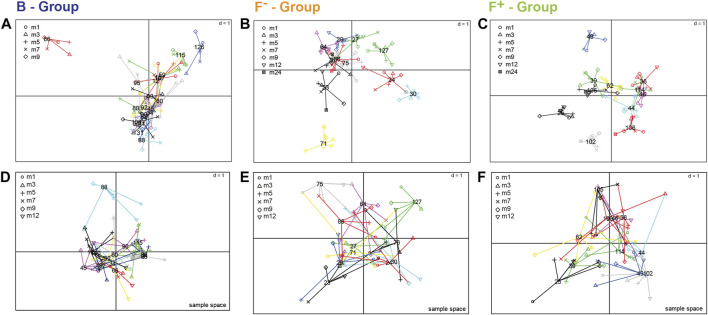
Sample spaces of multiple co-inertia analysis to visualize inter- and intra-individual differences over time, shown for the whole fecal polar metabolome screened with HILIC LC-MS approach **(A–C)** and for short chain fatty acids as selected microbial metabolites **(D–F)**. Each individual (number) is projected within its corresponding feeding group; **(A,D)** breastfed, **(B,E)** formula-fed without probiotics and **(C,F)** formula-fed with probiotics. For the exclusively breastfed group **(A)** only months 1–9 were considered because of a reduced number of available samples due to weaning. Samples from the same individual are linked by edges and the shapes represent the different time points. The shorter the edge, the higher the similarity of samples from the same individual. The more similar the metabolic profiles of individuals were the closer the projection in the sample space.

Longitudinal studies in microbiome research often described highly inter- and intra-individual variability (Zhou et al., 2019). The metabolite profiling of stool samples over time enables to determine the variability of each individual and metabolites that are responsible for this behavior. The inter- and intra-individual behavior of the children was elaborated by a multiple co-inertia analysis for the fecal polar metabolome, analyzed by HILIC LC-MS/MS (Figure 5; Supplementary Figure S6) (Meng et al., 2014). For the exclusively breastfed group (B) only months 1–9 were considered because of a reduced number of available samples in later months due to weaning. In the breastfed group (Figure 5A; Supplementary Table S5), especially infant 66 showed very individual profiles at certain time points. Metabolites responsible for this behavior were for example 3-hydroxyphenylacetic acid in month 5, taurocholic acid in month 7 (Supplementary Figure S7A) and N-acetylneuraminic acid in month 9. Infant 126 and 115 were characterized by FL in month 3 and ferulic acid sulfate in month 7. In the F- group (Figure 5B; Supplementary Table S5) the individuality was less distinct. For infant 71 hydroxydodecanoic acid (Supplementary Figure S7B) and polyphenolic compounds can be found in month 9 and 12, respectively. The individual profile of infant 30 is characterized e.g. by pantothenol in month 3, which is often used in care products, and fucose in month 9. In the F+ group (Figure 5C; Supplementary Table S5) infant 43 showed a different behavior over time due to e.g. cyclamic acid (Supplementary Figure S7C), an artificial sweetener in month three and 3-hydroxyphenylacetic acid in month 9, whereas infant 25 and 102 were characterized again by cyclamic acid in month 12 and phenyllactic acid in month 24. The more differentiated positions of the metabolites in the loading plot of group B and F+ (Supplementary Figure S6A,C) compared to the F- group (Supplementary Figure S6B) indicates a more pronounced individuality in group B and F+. For group B this could be explained by the fact that breast milk composition can be quite variable between mothers and over lactation periods (Villaseñor et al., 2014; Hascoët et al., 2019; Hewelt-Belka et al., 2019; John et al., 2019), whereas the formula-fed infants always received the same type of formula during the trial. The infant gut microbiota is influenced by the maternal diet during pregnancy, however, the effect of maternal diet during lactation is not understood so far (Sindi et al., 2021). Yet, in this study no data of maternal diet was collected, therefore no conclusions about the effect of mother’s diet on the infant microbiome and metabolome can be drawn. Additionally, in this study we did not collect data about the nature and frequency of food consumption in infants in later months of their early-life. Therefore, we cannot elaborate the origin of some individual metabolites such as the artificial sweetener cyclamic acid, ferulic acid sulfate or polyphenolic compounds. The individual behavior of the F+ group seemed to be more influenced by microbial metabolites. Wandro et al. reported that the inter-individual effect on the metabolome of infants over the first 6 weeks of life was stronger than any trends in clinical factors. Furthermore, for some individual infants strong shifts in the metabolite profiles were observed over time, while others remained more stable (Wandro et al., 2018). Not only the whole metabolome showed high intra- and inter-individual variability but also specific metabolite classes such short chain fatty acids (Mcorist et al., 2011). Infant 88 (B, Figure 5D), 75 (F-, Figure 5E), and 25 (F+, Figure 5F) showed the highest inter- and intra-individual behavior of SCFAs. High dispersion of lactic acid over time was observed in the loading plot of B group (Supplementary Figures S8A), but also for F- and F+ infants (Supplementary Figure S8, B-C). Butyric and propionic acid were also highly distributed over different time points in F- an F+ infants. Valeric, isovaleric and pyruvic showed a more closed distribution for all feeding groups. Only few infants of this study have been exposed to antibiotics (Supplrmentary Table S1). No aberrant microbiota profiles were seen for these individual time points, therefore no related effects on fecal metabolite profiles were assumed (Bazanella et al., 2017).

## Conclusion

**FIGURE 6:**
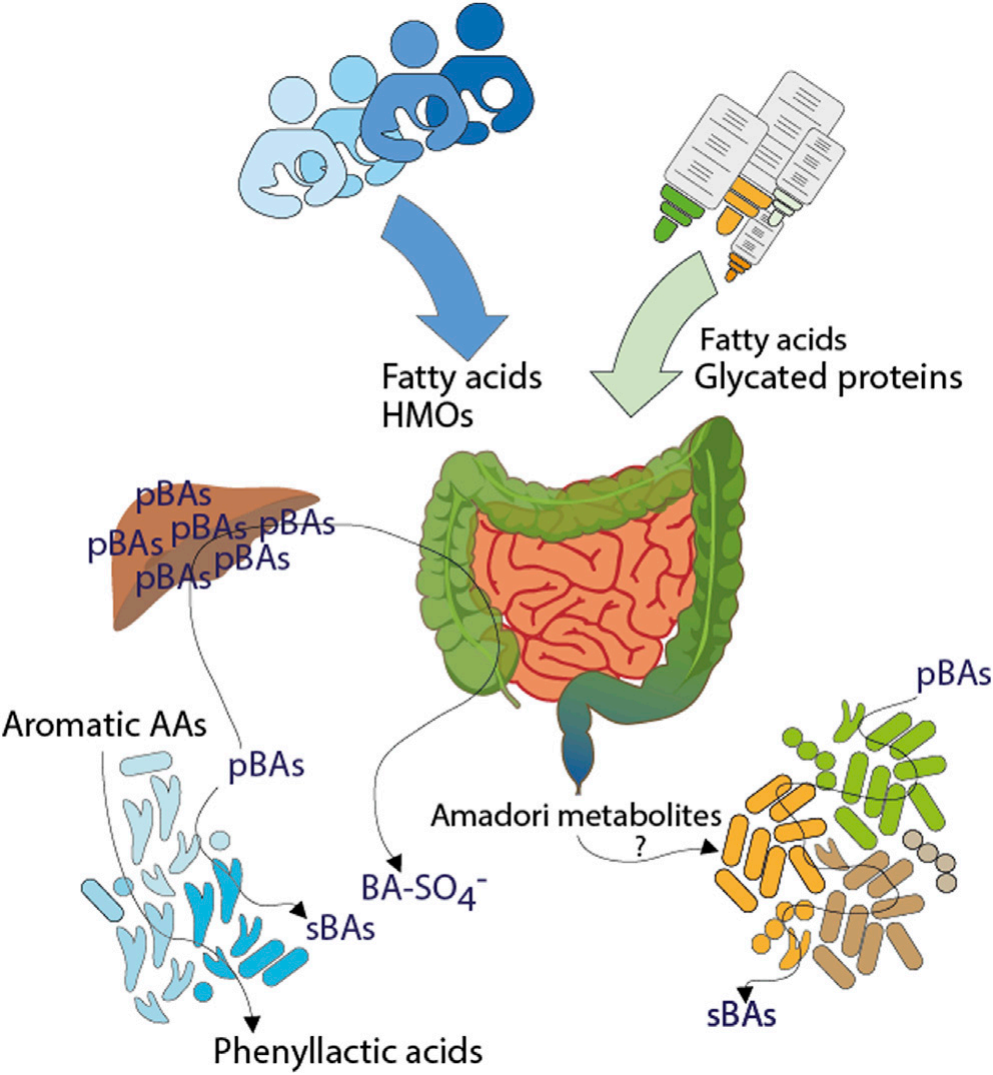
Influences of early life nutrition on the fecal metabolome of infants. Dietary ingredients, endogenous compounds but also microbial products are shaping the gut environment of breast- and formula-fed infants. Fatty acids, human milk oligosaccharides and glycated proteins are coming from diet, here breast milk or formula. During digestion, glycated proteins are degraded into small pieces, resulting in the excretion of Amadori metabolites or may influence the composition of gut microbiota. Aromatic amino acids are metabolized by resident gut microbiota in breast milk fed children resulting in the excretion of microbial phenyllactic acids. The infant diet could influence the synthesis of primary bile acids. The presence or absence of distinct microbiota is reflected by increased sulfated bile acids in breast milk fed infants and increased levels of secondary bile acids in formula fed infants. (pBAs = primary bile acids, sBAs = secondary bile acids, BA-SO_4_^−^ = bile acid sulfates, AAs = amino acids).

In summary, the fecal metabolite profiles of the infant cohort were largely composed of ingredients of consumed food and microbial metabolism of food compounds or microbial co-metabolism of host derived metabolites (Figure 6). Some of them are recovered directly without modification in stool such as long chain fatty acids or HMOs and may be directly linked to the child’s feeding type or mother’s diet or genotype. Furthermore, digested products of food processing (glycated proteins) were found especially in feces of formula-fed infants.

Altered microbial modification of amino acids or bile acids in breastfed and formula-fed infants resulted in distinct metabolite profiles, hinting toward different gut microbial populations and thus different microbial metabolism. It may reflect rather specialized or absent microbial metabolism for example of aromatic amino acids through bifidobacteria or increased sulfation of bile acids in breastfed children, respectively. Diverse microbial metabolism was illustrated by several secondary bile acids in formula-fed children. Overall, each individual was reflected by its own fecal metabolite profile and particular metabolites displayed high inter- and individual variability, induced by different genetic backgrounds or external influences, like the individual composition of breast milk, the amount of consumed formula or living environment.

## Data Availability

Raw sequence data are available at the European Nucleotide Archive under study accession number ERP023432. Metabolomics data is available as supplementary tables of supporting information.

## Abbreviations

ACN, acetonitrile; CA, cholic acid; CA-S, Cholic acid 7-sulfate; CDCA, chenodeoxycholic acid; Da, Dalton; ESI, electrospray ionization; 2-FL, 2′-fucosyllactose; 3-FL, 3′-fucosyllactose; GCA, glycocholic acid; GCDCA, glychochenodeoxycholic acid; H_2_O, Water; HILIC, hydrophilic interaction liquid chromatography; HMOs, human milk oligosaccharides; LCA, lithocholic acid; MRM, multiple reaction monitoring mode; 7-oxoDCA, 7-oxodeoxycholic acid; OTU, operational taxonomic unit; PLS-DA, partial least squares discriminant analysis; rCCA, regularized canonical correlation analysis; ROC, receiver operating characteristic; RP, reversed-phase; SCFAs, short chain fatty acids; TCA, taurocholic acid; TCDCA, Taurochenodeoxycholic acid; TOF, Time of Flight; UHPLC-MS, Ultrahigh-performance liquid chromatography mass spectrometry; UDCA, ursodeoxycholic acid.

## References

[B1] Alnouti Y. (2009). Bile Acid Sulfation: A Pathway of Bile Acid Elimination and Detoxification. Toxicol. Sci. 108, 225–246. 10.1093/toxsci/kfn268 19131563

[B2] Aragozzini F. Ferrari A. Pacini N. Gualandris R. (1979). Indole-3-lactic Acid as a Tryptophan Metabolite Produced by Bifidobacterium Spp. Appl. Environ. Microbiol. 38, 544–546. 10.1128/aem.38.3.544-546.1979 533277 PMC243529

[B3] Bazanella M. Maier T. V. Clavel T. Lagkouvardos I. Lucio M. Maldonado-Gòmez M. X. (2017). Randomized Controlled Trial on the Impact of Early-Life Intervention with Bifidobacteria on the Healthy Infant Fecal Microbiota and Metabolome. Am. J. Clin. Nutr. 106, 1274–1286. 10.3945/ajcn.117.157529 28877893

[B4] Begley M. Hill C. Gahan C. G. M. (2006). Bile Salt Hydrolase Activity in Probiotics. Aem 72, 1729–1738. 10.1128/aem.72.3.1729-1738.2006 PMC1393245 16517616

[B5] Beloborodov N. V. Khodakova A. S. Bairamov I. T. Olenin A. Y. (2009). Microbial Origin of Phenylcarboxylic Acids in the Human Body. Biochem. Mosc. 74, 1350–1355. 10.1134/s0006297909120086 19961416

[B6] Beloborodova N. Bairamov I. Olenin A. Shubina V. Teplova V. Fedotcheva N. (2012). Effect of Phenolic Acids of Microbial Origin on Production of Reactive Oxygen Species in Mitochondria and Neutrophils. J. Biomed. Sci. 19, 89. 10.1186/1423-0127-19-89 23061754 PMC3503878

[B7] Bode L. (2012). Human Milk Oligosaccharides: Every Baby Needs a Sugar Mama. Glycobiology 22, 1147–1162. 10.1093/glycob/cws074 22513036 PMC3406618

[B8] Bridgman S. L. Azad M. B. Field C. J. Haqq A. M. Becker A. B. Mandhane P. J. (2017). Fecal Short-Chain Fatty Acid Variations by Breastfeeding Status in Infants at 4 Months: Differences in Relative versus Absolute Concentrations. Front. Nutr. 4, 11. 10.3389/fnut.2017.00011 28443284 PMC5385454

[B9] Brink L. R. Mercer K. E. Piccolo B. D. Chintapalli S. V. Elolimy A. Bowlin A. K. (2020). Neonatal Diet Alters Fecal Microbiota and Metabolome Profiles at Different Ages in Infants Fed Breast Milk or Formula. Am. J. Clin. Nutr. 111, 1190–1202. 10.1093/ajcn/nqaa076 32330237 PMC7266684

[B10] Chow J. Panasevich M. R. Alexander D. Vester Boler B. M. Rossoni Serao M. C. Faber T. A. (2014). Fecal Metabolomics of Healthy Breast-Fed versus Formula-Fed Infants before and during *In Vitro* Batch Culture Fermentation. J. Proteome Res. 13, 2534–2542. 10.1021/pr500011w 24628373

[B11] De Leoz M. L. A. Gaerlan S. C. Strum J. S. Dimapasoc L. M. Mirmiran M. Tancredi D. J. (2012). Lacto-N-tetraose, Fucosylation, and Secretor Status Are Highly Variable in Human Milk Oligosaccharides from Women Delivering Preterm. J. Proteome Res. 11, 4662–4672. 10.1021/pr3004979 22900748 PMC3478894

[B12] Degirolamo C. Rainaldi S. Bovenga F. Murzilli S. Moschetta A. (2014). Microbiota Modification with Probiotics Induces Hepatic Bile Acid Synthesis via Downregulation of the Fxr-Fgf15 axis in Mice. Cel Rep. 7, 12–18. 10.1016/j.celrep.2014.02.032 24656817

[B13] Dodd D. Spitzer M. H. Van Treuren W. Merrill B. D. Hryckowian A. J. Higginbottom S. K. (2017). A Gut Bacterial Pathway Metabolizes Aromatic Amino Acids into Nine Circulating Metabolites. Nature 551, 648–652. 10.1038/nature24661 29168502 PMC5850949

[B14] Donazzolo E. Gucciardi A. Mazzier D. Peggion C. Pirillo P. Naturale M. (2017). Improved Synthesis of glycine, Taurine and Sulfate Conjugated Bile Acids as Reference Compounds and Internal Standards for ESI-MS/MS Urinary Profiling of Inborn Errors of Bile Acid Synthesis. Chem. Phys. Lipids 204, 43–56. 10.1016/j.chemphyslip.2017.03.004 28300538

[B15] Dotz V. Adam R. Lochnit G. Schroten H. Kunz C. (2016). Neutral Oligosaccharides in Feces of Breastfed and Formula-Fed Infants at Different Ages. Glycobiology 26, 1308–1316. 10.1093/glycob/cww087 27613801

[B16] Dotz V. Rudloff S. Meyer C. Lochnit G. Kunz C. (2015). Metabolic Fate of Neutral Human Milk Oligosaccharides in Exclusively Breast-Fed Infants. Mol. Nutr. Food Res. 59, 355–364. 10.1002/mnfr.201400160 25330044

[B17] Eyssen H. (1973). Role of the Gut Microflora in Metabolism of Lipids and Sterols. Proc. Nutr. Soc. 32, 59–63. 10.1079/pns19730016 4598411

[B18] Fiorucci S. Distrutti E. (2015). Bile Acid-Activated Receptors, Intestinal Microbiota, and the Treatment of Metabolic Disorders. Trends Mol. Med. 21, 702–714. 10.1016/j.molmed.2015.09.001 26481828

[B19] Firl N. Kienberger H. Rychlik M. (2014). Validation of the Sensitive and Accurate Quantitation of the Fatty Acid Distribution in Bovine Milk. Int. Dairy J. 35, 139–144. 10.1016/j.idairyj.2013.11.007

[B20] González I. Cao K.-a. L. Davis M. J. Déjean S. (2012). Visualising Associations between Paired 'omics' Data Sets. BioData mining 5, 19. 10.1186/1756-0381-5-19 23148523 PMC3630015

[B21] González I. Déjean S. Martin P. G. P. Baccini A. (2008). CCA: An R Package to Extend Canonical Correlation Analysis. J. Stat. Softw. 23, 14. 10.18637/jss.v023.i12

[B22] Grill J. P. Manginot-Dürr C. Schneider F. Ballongue J. (1995). Bifidobacteria and Probiotic Effects: Action of Bifidobacterium Species on Conjugated Bile Salts. Curr. Microbiol. 31, 23–27. 10.1007/bf00294629 7767225

[B23] Hammons J. L. Jordan W. E. Stewart R. L. Taulbee J. D. Berg R. W. (1988). Age and Diet Effects on Fecal Bile Acids in Infants. J. Pediatr. Gastroenterol. Nutr. 7, 30–38. 10.1097/00005176-198801000-00008 3335983

[B24] Hascoët J.-M. Chauvin M. Pierret C. Skweres S. Egroo L.-D. V. Rougé C. (2019). Impact of Maternal Nutrition and Perinatal Factors on Breast Milk Composition after Premature Delivery. Nutrients 11, 366. 10.3390/nu11020366 PMC6413091 30744155

[B25] He X. Parenti M. Grip T. Lönnerdal B. Timby N. Domellöf M. (2019). Fecal Microbiome and Metabolome of Infants Fed Bovine MFGM Supplemented Formula or Standard Formula with Breast-Fed Infants as Reference: a Randomized Controlled Trial. Scientific Rep. 9, 11589. 10.1038/s41598-019-47953-4 PMC6690946 31406230

[B26] Hegele J. Buetler T. Delatour T. (2008). Comparative LC-MS/MS Profiling of Free and Protein-Bound Early and Advanced Glycation-Induced Lysine Modifications in Dairy Products. Analytica Chim. Acta 617, 85–96. 10.1016/j.aca.2007.12.027 18486644

[B27] Henning C. Smuda M. Girndt M. Ulrich C. Glomb M. A. (2011). Molecular Basis of Maillard Amide-Advanced Glycation End Product (AGE) Formation *In Vivo* . J. Biol. Chem. 286, 44350–44356. 10.1074/jbc.m111.282442 22069309 PMC3248017

[B28] Heubi J. E. Balistreri W. F. Suchy F. J. (1982). Bile Salt Metabolism in the First Year of Life. J. Lab. Clin. Med. 100, 127–136. 7201000

[B29] Hewelt-Belka W. Garwolińska D. Belka M. Bączek T. Namieśnik J. Kot-Wasik A. (2019). A New Dilution-Enrichment Sample Preparation Strategy for Expanded Metabolome Monitoring of Human Breast Milk that Overcomes the Simultaneous Presence of Low- and High-Abundance Lipid Species. Food Chem. 288, 154–161. 10.1016/j.foodchem.2019.03.001 30902276

[B30] Huang C. T. L. Rodriguez J. T. Woodward W. E. Nichols B. L. (1976). Comparison of Patterns of Fecal Bile Acid and Neutral Sterol between Children and Adults. Am. J. Clin. Nutr. 29, 1196–1203. 10.1093/ajcn/29.11.1196 793370

[B31] John A. Sun R. Maillart L. Schaefer A. Hamilton Spence E. Perrin M. T. (2019). Macronutrient Variability in Human Milk from Donors to a Milk Bank: Implications for Feeding Preterm Infants. PLOS ONE 14, e0210610. 10.1371/journal.pone.0210610 30682200 PMC6347243

[B32] Kim G.-B. Miyamoto C. M. Meighen E. A. Lee B. H. (2004). Cloning and Characterization of the Bile Salt Hydrolase Genes (Bsh) from Bifidobacterium Bifidum Strains. Aem 70, 5603–5612. 10.1128/aem.70.9.5603-5612.2004 PMC520925 15345449

[B33] Kim H. Kang S. Jung B.-M. Yi H. Jung J. A. Chang N. (2017). Breast Milk Fatty Acid Composition and Fatty Acid Intake of Lactating Mothers in South Korea. Br. J. Nutr. 117, 556–561. 10.1017/s0007114517000253 28285609

[B34] Kok C. R. Brabec B. Chichlowski M. Harris C. L. Moore N. Wampler J. L. (2020). Stool Microbiome, pH and Short/branched Chain Fatty Acids in Infants Receiving Extensively Hydrolyzed Formula, Amino Acid Formula, or Human Milk through Two Months of Age. BMC Microbiol. 20, 337. 10.1186/s12866-020-01991-5 33167908 PMC7650147

[B35] Koletzko B. Baker S. Cleghorn G. Neto U. F. Gopalan S. Hernell O. (2005). Global Standard for the Composition of Infant Formula: Recommendations of an ESPGHAN Coordinated International Expert Group. J. Pediatr. Gastroenterol. Nutr. 41, 584–599. 10.1097/01.mpg.0000187817.38836.42 16254515

[B36] Lagkouvardos I. Joseph D. Kapfhammer M. Giritli S. Horn M. Haller D. (2016). IMNGS: A Comprehensive Open Resource of Processed 16S rRNA Microbial Profiles for Ecology and Diversity Studies. Scientific Rep. 6, 33721. 10.1038/srep33721 PMC5034312 27659943

[B37] Laurens M. L. L. Kraus-Friedberg C. Kar W. Sanfilippo D. Rajasekaran S. Comstock S. S. (2020). Dietary Intake Influences Metabolites in Healthy Infants: A Scoping Review. Nutrients 12, 2073. 10.3390/nu12072073 PMC7400847 32668684

[B38] Lester R. Pyrek J. S. Little J. M. Adcock E. W. (1983). Diversity of Bile Acids in the Fetus and Newborn Infant. J. Pediatr. Gastroenterol. Nutr. 2, 355–364. 10.1097/00005176-198302020-00026 6348233

[B39] Lewis Z. T. Totten S. M. Smilowitz J. T. Popovic M. Parker E. Lemay D. G. (2015). Maternal Fucosyltransferase 2 Status Affects the Gut Bifidobacterial Communities of Breastfed Infants. Microbiome 3, 13. 10.1186/s40168-015-0071-z 25922665 PMC4412032

[B40] Li N. Yan F. Wang N. Song Y. Yue Y. Guan J. (2020). Distinct Gut Microbiota and Metabolite Profiles Induced by Different Feeding Methods in Healthy Chinese Infants. Front. Microbiol. 11, 714. 10.3389/fmicb.2020.00714 32435235 PMC7219020

[B41] Martin C. Ling P.-R. Blackburn G. (2016a). Review of Infant Feeding: Key Features of Breast Milk and Infant Formula. Nutrients 8, 279. 10.3390/nu8050279 PMC4882692 27187450

[B42] Martin F.-P. J. Moco S. Montoliu I. Collino S. Da Silva L. Rezzi S. (2014). Impact of Breast-Feeding and High- and Low-Protein Formula on the Metabolism and Growth of Infants from Overweight and Obese Mothers. Pediatr. Res. 75, 535–543. 10.1038/pr.2013.250 24375085

[B43] Martin R. Makino H. Cetinyurek Yavuz A. Ben-Amor K. Roelofs M. Ishikawa E. (2016b). Early-Life Events, Including Mode of Delivery and Type of Feeding, Siblings and Gender, Shape the Developing Gut Microbiota. PLOS ONE 11, e0158498. 10.1371/journal.pone.0158498 27362264 PMC4928817

[B44] Mcorist A. L. Miller R. B. Bird A. R. Keogh J. B. Noakes M. Topping D. L. (2011). Fecal Butyrate Levels Vary Widely Among Individuals but Are Usually Increased by a Diet High in Resistant Starch. J. Nutr. 141, 883–889. 10.3945/jn.110.128504 21430242

[B45] Meng C. Kuster B. Culhane A. C. Gholami A. (2014). A Multivariate Approach to the Integration of Multi-Omics Datasets. BMC Bioinformatics 15, 162. 10.1186/1471-2105-15-162 24884486 PMC4053266

[B46] Mercer K. E. Bhattacharyya S. Diaz-Rubio M. E. Piccolo B. D. Pack L. M. Sharma N. (2018). Infant Formula Feeding Increases Hepatic Cholesterol 7α Hydroxylase (CYP7A1) Expression and Fecal Bile Acid Loss in Neonatal Piglets. J. Nutr. 148, 702–711. 10.1093/jn/nxy038 30053282 PMC6857617

[B47] Neelima Sharma R. Rajput Y. S. Mann B. (2013). Chemical and Functional Properties of Glycomacropeptide (GMP) and its Role in the Detection of Cheese Whey Adulteration in Milk: a Review. Dairy Sci. Technol. 93, 21–43. 10.1007/s13594-012-0095-0 23396893 PMC3567326

[B48] Pischetsrieder M. Henle T. (2012). Glycation Products in Infant Formulas: Chemical, Analytical and Physiological Aspects. Amino Acids 42, 1111–1118. 10.1007/s00726-010-0775-0 20953645

[B49] Plows J. F. Berger P. K. Jones R. B. Alderete T. L. Yonemitsu C. Najera J. A. (2021). Longitudinal Changes in Human Milk Oligosaccharides (HMOs) over the Course of 24 Months of Lactation. J. Nutr. 151, 876–882. 10.1093/jn/nxaa427 33693851 PMC8030713

[B50] Poroyko V. Morowitz M. Bell T. Ulanov A. Wang M. Donovan S. (2011). Diet Creates Metabolic Niches in the "immature Gut" that Shape Microbial Communities. Nutr. Hosp. 26, 1283–1295. 10.1590/S0212-16112011000600015 22411374

[B51] Pranger I. G. Corpeleijn E. Muskiet F. A. J. Kema I. P. Singh-Povel C. Bakker S. J. L. (2019). Circulating Fatty Acids as Biomarkers of Dairy Fat Intake: Data from the Lifelines Biobank and Cohort Study. Biomarkers 24, 360–372. 10.1080/1354750x.2019.1583770 30773031

[B52] Prentice A. (1996). Constituents of Human Milk. Food Nutr. Bull. 17, 1–10. 10.1177/156482659601700406

[B53] Remoroza C. A. Liang Y. Mak T. D. Mirokhin Y. Sheetlin S. L. Yang X. (2020). Increasing the Coverage of a Mass Spectral Library of Milk Oligosaccharides Using a Hybrid-Search-Based Bootstrapping Method and Milks from a Wide Variety of Mammals. Anal. Chem. 92, 10316–10326. 10.1021/acs.analchem.0c00342 32639750 PMC10939002

[B54] Remoroza C. A. Mak T. D. De Leoz M. L. A. Mirokhin Y. A. Stein S. E. (2018). Creating a Mass Spectral Reference Library for Oligosaccharides in Human Milk. Anal. Chem. 90, 8977–8988. 10.1021/acs.analchem.8b01176 29969231

[B55] Rigo J. Boehm G. Georgi G. Jelinek J. Nyambugabo K. Sawatzki G. (2001). An Infant Formula Free of Glycomacropeptide Prevents Hyperthreoninemia in Formula-Fed Preterm Infants. J. Pediatr. Gastroenterol. Nutr. 32, 127–130. 10.1097/00005176-200102000-00006 11321379

[B56] Robinson S. M. (2015). Infant Nutrition and Lifelong Health: Current Perspectives and Future Challenges. J. Dev. Orig Health Dis. 6, 384–389. 10.1017/s2040174415001257 26088394 PMC4634203

[B57] Rosa F. Matazel K. S. Bowlin A. K. Williams K. D. Elolimy A. A. Adams S. H. (2020). Neonatal Diet Impacts the Large Intestine Luminal Metabolome at Weaning and Post-Weaning in Piglets Fed Formula or Human Milk. Front. Immunol. 11, 3167. 10.3389/fimmu.2020.607609 PMC7750455 33365033

[B58] Ruan D. Wang H. Cheng F. (2018). The Maillard Reaction in Food Chemistry: Current Technology and Applications. Springer Briefs in Molecular Science. Cham, Switzerland: Springer. 10.1007/978-3-030-04777-1

[B59] Sillner N. Walker A. Harrieder E.-M. Schmitt-Kopplin P. Witting M. (2019a). Development and Application of a HILIC UHPLC-MS Method for Polar Fecal Metabolome Profiling. J. Chromatogr. B 1109, 142–148. 10.1016/j.jchromb.2019.01.016 30763867

[B60] Sillner N. Walker A. Hemmler D. Bazanella M. Heinzmann S. S. Haller D. (2019b). Milk-Derived Amadori Products in Feces of Formula-Fed Infants. J. Agric. Food Chem. 67, 8061–8069. 10.1021/acs.jafc.9b01889 31264412

[B61] Sillner N. Walker A. Koch W. Witting M. Schmitt-Kopplin P. (2018). Metformin Impacts Cecal Bile Acid Profiles in Mice. J. Chromatogr. B 1083, 35–43. 10.1016/j.jchromb.2018.02.029 29522956

[B62] Sindi A. S. Geddes D. T. Wlodek M. E. Muhlhausler B. S. Payne M. S. Stinson L. F. (2021). Can We Modulate the Breastfed Infant Gut Microbiota through Maternal Diet? FEMS Microbiol. Rev., fuab011. 10.1093/femsre/fuab011 33571360

[B63] Smuda M. Voigt M. Glomb M. A. (2010). Degradation of 1-Deoxy-D-Erythro-Hexo-2,3-Diulose in the Presence of Lysine Leads to Formation of Carboxylic Acid Amides. J. Agric. Food Chem. 58, 6458–6464. 10.1021/jf100334r 20429584

[B64] Sperisen P. Cominetti O. Martin F.-P. (2015). Longitudinal Omics Modeling and Integration in Clinical Metabonomics Research: Challenges in Childhood Metabolic Health Research. Front. Mol. Biosciences 2, 44. 10.3389/fmolb.2015.00044 PMC4525019 26301225

[B65] Stewart C. J. Ajami N. J. O’Brien J. L. Hutchinson D. S. Smith D. P. Wong M. C. (2018). Temporal Development of the Gut Microbiome in Early Childhood from the TEDDY Study. Nature 562, 583–588. 10.1038/s41586-018-0617-x 30356187 PMC6415775

[B66] Sumner L. W. Amberg A. Barrett D. Beale M. H. Beger R. Daykin C. A. (2007). Proposed Minimum Reporting Standards for Chemical Analysis. Metabolomics 3, 211–221. 10.1007/s11306-007-0082-2 24039616 PMC3772505

[B67] Tanaka H. Hashiba H. Kok J. Mierau I. (2000). Bile Salt Hydrolase of Bifidobacterium Longum-Biochemical and Genetic Characterization. Appl. Environ. Microbiol. 66, 2502–2512. 10.1128/aem.66.6.2502-2512.2000 10831430 PMC110569

[B68] Van Den Elsen L. W. J. Garssen J. Burcelin R. Verhasselt V. (2019). Shaping the Gut Microbiota by Breastfeeding: The Gateway to Allergy Prevention? Front. Pediatr. 747. 10.3389/fped.2019.00047 PMC6400986 30873394

[B69] Villaseñor A. Garcia-Perez I. Garcia A. Posma J. M. Fernández-López M. Nicholas A. J. (2014). Breast Milk Metabolome Characterization in a Single-phase Extraction, Multiplatform Analytical Approach. Anal. Chem. 86, 8245–8252. 10.1021/ac501853d 25058331

[B70] Wandro S. Osborne S. Enriquez C. Bixby C. Arrieta A. Whiteson K. (2018). The Microbiome and Metabolome of Preterm Infant Stool Are Personalized and Not Driven by Health Outcomes, Including Necrotizing Enterocolitis and Late-Onset Sepsis. mSphere 3, e00104. 10.1128/msphere.00104-18 29875143 PMC5990886

[B71] Wang J. Lu Y.-M. Liu B.-Z. He H.-Y. (2008). Electrospray Positive Ionization Tandem Mass Spectrometry of Amadori Compounds. J. Mass. Spectrom. 43, 262–264. 10.1002/jms.1290 17935069

[B72] Wang Q. Garrity G. M. Tiedje J. M. Cole J. R. (2007). Naïve Bayesian Classifier for Rapid Assignment of rRNA Sequences into the New Bacterial Taxonomy. Aem 73, 5261–5267. 10.1128/aem.00062-07 PMC1950982 17586664

[B73] Wild J. Shanmuganathan M. Hayashi M. Potter M. Britz-Mckibbin P. (2019). Metabolomics for Improved Treatment Monitoring of Phenylketonuria: Urinary Biomarkers for Non-invasive Assessment of Dietary Adherence and Nutritional Deficiencies. Analyst 144, 6595–6608. 10.1039/c9an01642b 31608347

[B74] Wishart D. S. Feunang Y. D. Marcu A. Guo A. C. Liang K. Vázquez-Fresno R. (2018). HMDB 4.0: the Human Metabolome Database for 2018. Nucleic Acids Res. 46, D608–d617. 10.1093/nar/gkx1089 29140435 PMC5753273

[B75] Wolf A. R. Wesener D. A. Cheng J. Houston-Ludlam A. N. Beller Z. W. Hibberd M. C. (2019). Bioremediation of a Common Product of Food Processing by a Human Gut Bacterium. Cell Host & Microbe 26, 463–477. 10.1016/j.chom.2019.09.001 31585844 PMC6801109

[B76] World Health Organization (2003). Global Strategy for Infant and Young Child Feeding. Geneva, Switzerland: World Health Organization.

[B77] Xiang M. Harbige L. Zetterström R. (2005). Long-chain Polyunsaturated Fatty Acids in Chinese and Swedish Mothers: Diet, Breast Milk and Infant Growth. Acta Paediatr. 94, 1543–1549. 10.1080/08035250500251601 16303692

[B78] Zhou W. Sailani M. R. Contrepois K. Zhou Y. Ahadi S. Leopold S. R. (2019). Longitudinal Multi-Omics of Host-Microbe Dynamics in Prediabetes. Nature 569, 663–671. 10.1038/s41586-019-1236-x 31142858 PMC6666404

[B79] Zierer J. Jackson M. A. Kastenmüller G. Mangino M. Long T. Telenti A. (2018). The Fecal Metabolome as a Functional Readout of the Gut Microbiome. Nat. Genet. 50, 790–795. 10.1038/s41588-018-0135-7 29808030 PMC6104805

