# Supplementary material for: Longitudinal Profiles of Dietary and Microbial Metabolites in Formula- and Breastfed Infants
Source: Front Mol Biosci. 2021 May 28;8:660456. doi: 10.3389/fmolb.2021.660456 (PMC8195334; doi:10.3389/fmolb.2021.660456)
Supplement: Supplementary file 6 [file datasheet1.docx]

**Supplementary Information**

**Longitudinal profiles of dietary and microbial metabolites in formula- and breastfed infants**

Nina Sillner^1,2^, Alesia Walker^1*^, Marianna Lucio^1^, Tanja V Maier^1^, Monika Bazanella^3^, Michael Rychlik^4^, Dirk Haller^2,3^, Philippe Schmitt-Kopplin^1,2,4^

^1^Research Unit Analytical BioGeoChemistry, Helmholtz Zentrum München, Neuherberg, Germany

^2^ZIEL Institute for Food and Health, Technical University of Munich, Freising, Germany

^3^Chair of Nutrition and Immunology, Technical University of Munich, Freising, Germany

^4^Chair of Analytical Food Chemistry, Technical University of Munich, Freising, Germany

*Corresponding author

E-Mail: alesia.walker@helmholtz-muenchen.de

HelmholtzZentrum München

Deutsches Forschungszentrum für Gesundheit und Umwelt

Department of Environmental Sciences

Analytical BioGeoChemistry

Ingolstaedter Landstrasse 1

85764 Neuherberg

Germany

Tel: +49 89 3187 4656

**Supplementary figures**

**
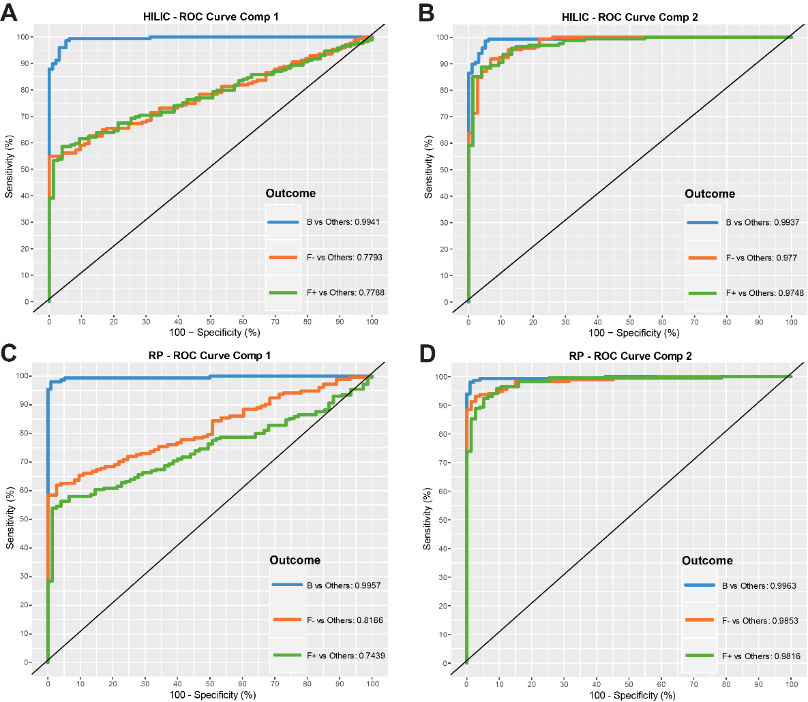
**

**Figure S1.** ROC (Receiver Operating Characteristics) curves from the multilevel PLS-DA of (A) HILIC principle component 1, (B) HILIC principle component 2, (C) RP principle component 1 and (D) RP principle component 2 UHPLC-MS measurements (negative ionization mode). The area under the curves indicate a very good separation of breastfed (B) versus formula-fed (F- and F+) infants for both data sets in principle component 1 and 2. Less valid differences between F- and F+ were found in component 2.


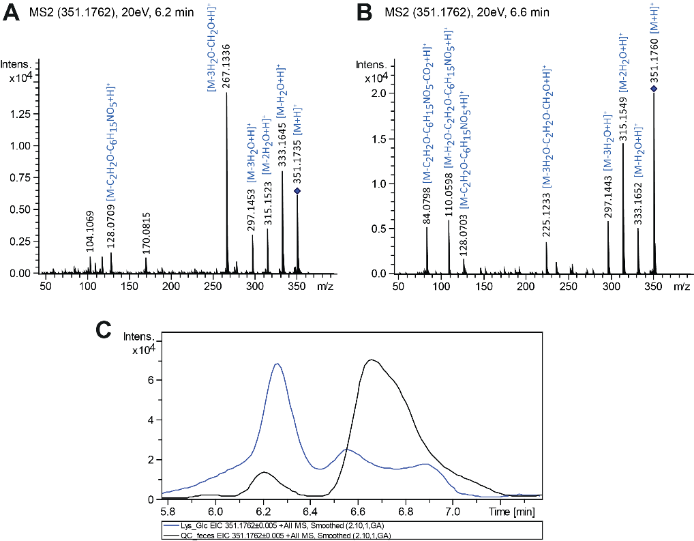


**Figure S2.** Collision induced dissociation MS/MS experiments (20 eV, positive ionization mode) of the putative Amadori product FruAcLys isomers with (A) retention time at 6.2 min and (B) 6.6 min, measured with HILIC UHPLC-MS, which were found to be significantly increased in formula-fed infants. (C) Overlaid extracted ion chromatograms of FruAcLys ([M+H]^+^ = 351.1762 ± 0.005 Da) in feces (black) and in a model reaction mixture of L-lysine with glucose, heated for 1 h at 100 °C in water (blue).

**
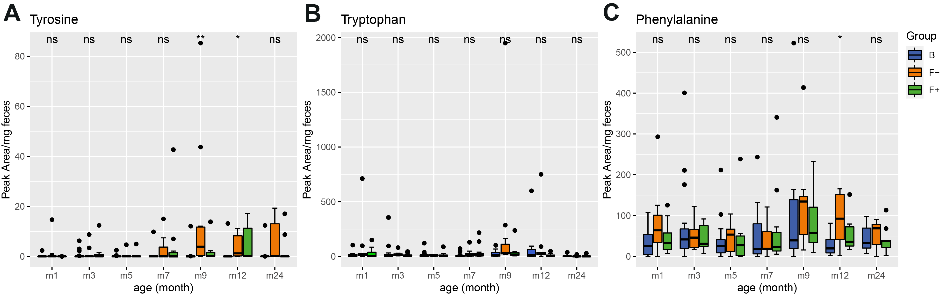
**

**Figure S3.** Profiles of the aromatic amino acids (A) tyrosine, (B) tryptophan and (C) phenylalanine in feces of breastfed (group B) and formula-fed infants without (group F-) or with probiotics (group F+) over time. Significance was calculated in each month between B, F- and F+ group using the Kruskal-Wallis rank sum test. ns: p > 0.05, *: p ≤ 0.05, **: p ≤ 0.01, ***: p ≤ 0.001, ****: p ≤ 0.0001.


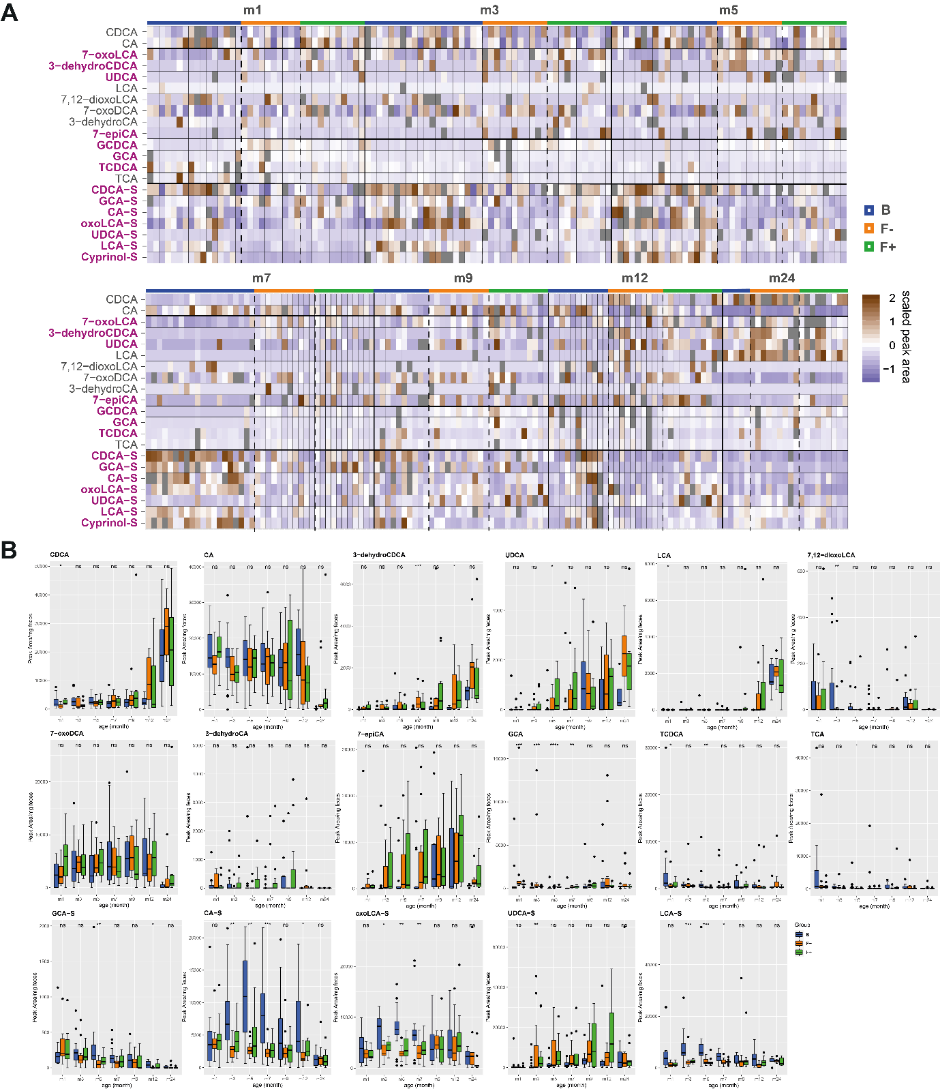


**Figure S4**: (A) Heatmap of all 21 detected bile acids in feces of breastfed (B, blue) and formula-fed infants without (F-, orange) or with probiotics (F+, green) over time. Peak areas (RP-UHPLC-MS) were unit variance scaled. Scaled peak areas > 2 were removed and displayed grey for better visualization. Bile acids which were altered due to the different diets (derived from multilevel PLS-DA) are marked purple. (B) Individual profiles of 17 bile acids detected in fecal samples of breastfed or formula milk fed infants. Significance was calculated in each month between B, F- and F+ group using the Kruskal-Wallis rank sum test. ns: p > 0.05, *: p ≤ 0.05, **: p ≤ 0.01, ***: p ≤ 0.001, ****: p ≤ 0.0001.


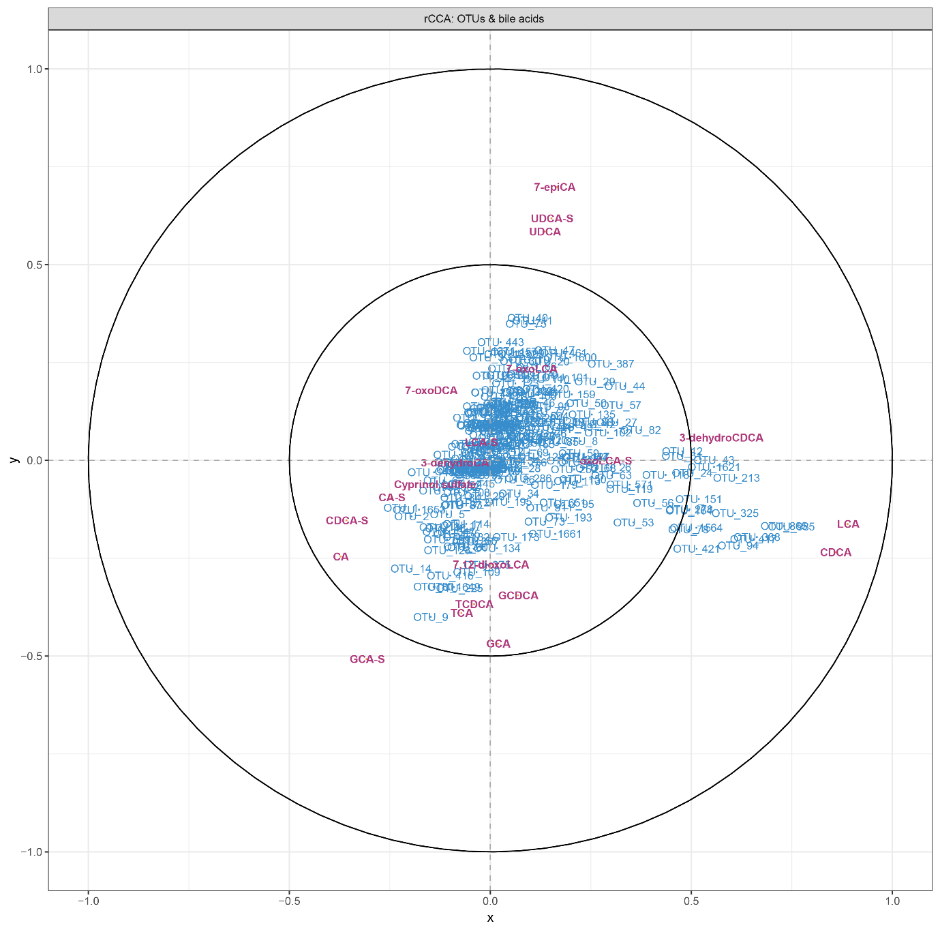
**Figure S5.** Representation of variables defined by the first two canonical variates from a regularized canonical correlation analysis (rCCA) of bile acids and OTU data from 16S rRNA sequencing detected in feces samples from all infants. Bile acids are colored in purple and OTUs in blue.


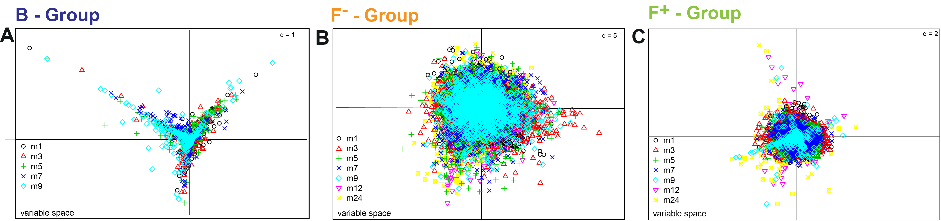


**Figure S6.** Variable spaces of multiple co-inertia analysis to visualize inter- and intra-individual differences over time, separated for feeding groups; (A) breastfed (B), (B) formula-fed without probiotics (F-) and (C) formula-fed with probiotics (F+). Shapes and colors represent the different time points. For group B only months 1 – 9 were taken into account because of a reduced number of available samples in later months due to weaning.


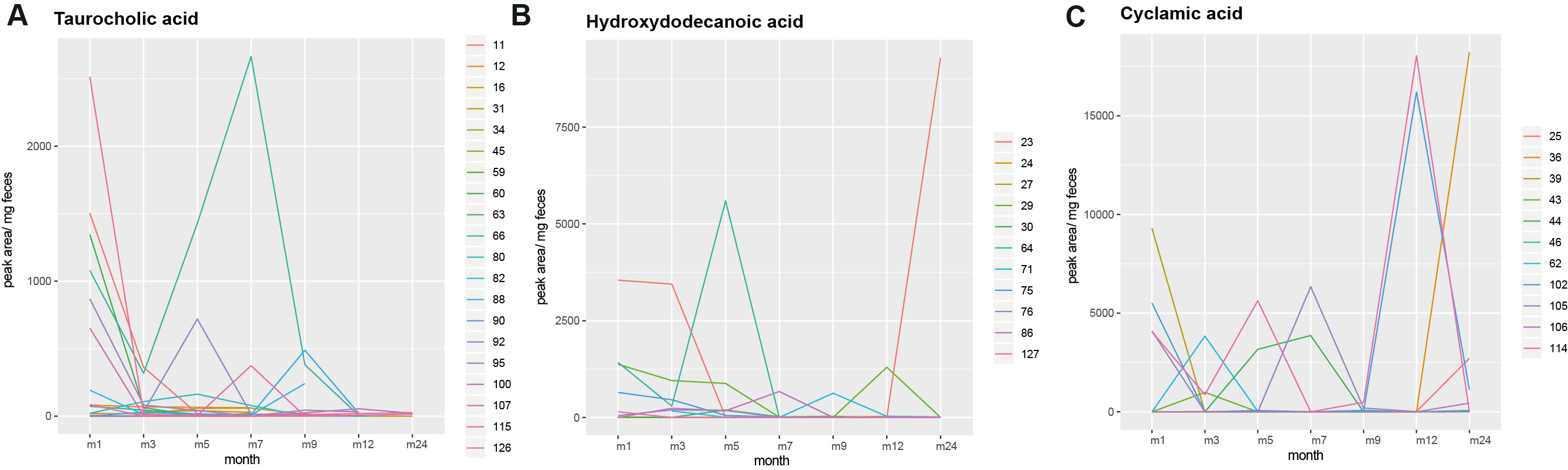


**Figure S7.** Inter- and intra-individual profiles of three selected metabolites. (A) Increased value of taurocholic acid was found in infant 66 (breastfed) at month 7. (B) High amount of hydroxydodecanoic acid in infant 71 (F-) at month 9. (C) High signals of cyclamic acid were detected in infant 25 and 102 (both F+) at month 12.


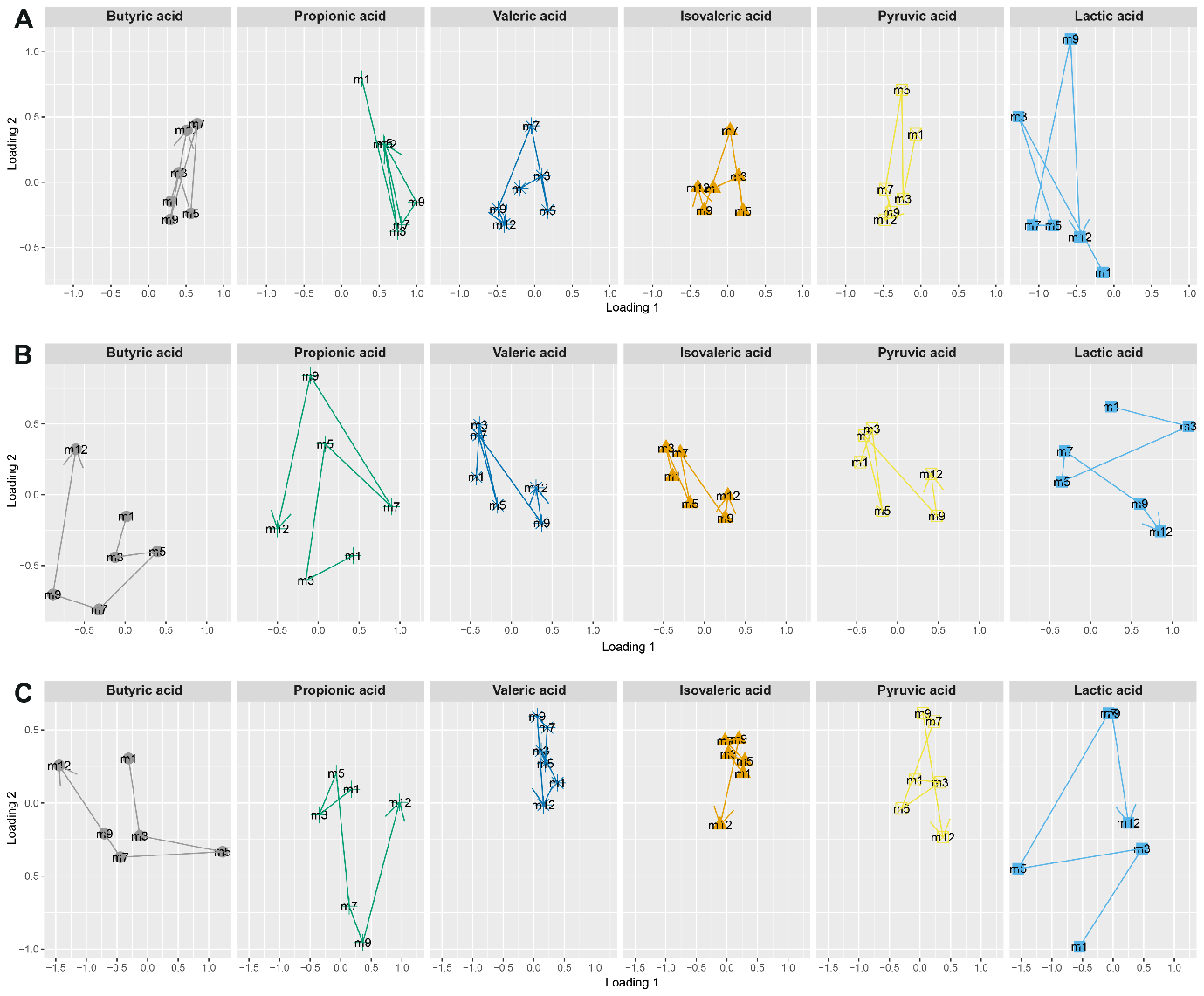


**Figure S8.** Loading plots of multiple co-inertia analysis of six short chain fatty acids (SCFAs) for breastfed (A), F- (B) and F+ (C), illustrating the dispersion of SCFAs over time. Lactic acid showed high inter- and intra-individual variability in all three groups. Butyric and propionic acid were also highly variable in F- and F+ infants.

# Data availability

**Identification of RP LC-MS features on selected samples:**

A) 46 samples selected on ID match from 244 samples on RP (Figure 1A)

MassIVE MSV000086026

B) Fatty acid matching (Figure 2A-C) by MS1 (±0.005Da) and retention time

MassIVE MSV000086031

C) Bile acid matching (Figure 4A-C) by MS1 (±0.005Da) and retention time

MassIVE MSV000086032

**Identification of HILIC LC-MS features on all samples:**

HILIC LC-MS/MS of 244 fecal samples (n=2 replicates), (Figure 1B, Figure 3C-E)

10 eV and 35 eV

**Part I: 1_HILIC LC MS_MS of 244 fecal samples of infants_part I - 10 eV**

MassIVE MSV000086028

**Part II: 1_HILIC LC MS_MS of 244 fecal samples of infants_part II - 35 eV**

MassIVE MSV000086029

**Data for the elaboration of human milk oligosaccharides in feces of infants and breast milk samples (Figure 2D)**

2_HILIC LC_MS_MS of infant fecal samples and breast milk samples for analysis of HMOs_v1

MassIVE MSV000086030

**MS/MS of diverse Metabolites (add single annotated Bronze spectrum function)**

**Amadori Products**

MetAla-Glc ( CCMSLIB00005724018)

FruAcLys (2 Clusters – isomers)

α-acetyllysine; FruAcLys - Cluster_10927:

FruAcLys_RT6.2_1818_isomer1: (CCMSLIB00005724023)

ε-acetyllysine; FruAcLys - Cluster_10929:

FruAcLys_RT6.6_1943_isomer2: (CCMSLIB00005724021)

**Bile acids identification – sulfate conjugates**

UDCA-S (CCMSLIB00005720321)

LCA-S (CCMSLIB00005720905)

CDCA-S (CCMSLIB00005723982)

CA-S (CCMSLIB00005724014)
